# New insights into the evolutionary history of plant sorbitol dehydrogenase

**DOI:** 10.1186/s12870-015-0478-5

**Published:** 2015-04-12

**Authors:** Yong Jia, Darren CJ Wong, Crystal Sweetman, John B Bruning, Christopher M Ford

**Affiliations:** School of Agriculture, Food and Wine, University of Adelaide, Adelaide, 5005 Australia; Present address: Wine Research Center, Faculty of Land and Food Systems, University of British Columbia, Vancouver, V6T 1Z4 BC Canada; Present address: School of Biological Sciences, Flinders University, GPO Box 2100, Adelaide, 5001 Australia; School of Biological Sciences, University of Adelaide, Adelaide, 5005 Australia

**Keywords:** Sorbitol dehydrogenase, L-idonate-5-dehydrogenase, Gene duplication, Functional divergence, Tartaric acid, Ascorbic acid, Grapevine

## Abstract

**Background:**

Sorbitol dehydrogenase (SDH, EC 1.1.1.14) is the key enzyme involved in sorbitol metabolism in higher plants. SDH genes in some *Rosaceae* species could be divided into two groups. L-idonate-5-dehydrogenase (LIDH, EC 1.1.1.264) is involved in tartaric acid (TA) synthesis in *Vitis vinifera* and is highly homologous to plant SDHs. Despite efforts to understand the biological functions of plant SDH, the evolutionary history of plant SDH genes and their phylogenetic relationship with the *V. vinifera* LIDH gene have not been characterized.

**Results:**

A total of 92 SDH genes were identified from 42 angiosperm species. SDH genes have been highly duplicated within the *Rosaceae* family while monocot, *Brassicaceae* and most *Asterid* species exhibit singleton SDH genes. Core Eudicot SDHs have diverged into two phylogenetic lineages, now classified as SDH Class I and SDH Class II. *V. vinifera* LIDH was identified as a Class II SDH. Tandem duplication played a dominant role in the expansion of plant SDH family and Class II SDH genes were positioned in tandem with Class I SDH genes in several plant genomes. Protein modelling analyses of *V. vinifera* SDHs revealed 19 putative active site residues, three of which exhibited amino acid substitutions between Class I and Class II SDHs and were influenced by positive natural selection in the SDH Class II lineage. Gene expression analyses also demonstrated a clear transcriptional divergence between Class I and Class II SDH genes in *V. vinifera* and *Citrus sinensis* (orange).

**Conclusions:**

Phylogenetic, natural selection and synteny analyses provided strong support for the emergence of SDH Class II by positive natural selection after tandem duplication in the common ancestor of core Eudicot plants. The substitutions of three putative active site residues might be responsible for the unique enzyme activity of *V. vinifera* LIDH, which belongs to SDH Class II and represents a novel function of SDH in *V. vinifera* that may be true also of other Class II SDHs. Gene expression analyses also supported the divergence of SDH Class II at the expression level. This study will facilitate future research into understanding the biological functions of plant SDHs.

**Electronic supplementary material:**

The online version of this article (doi:10.1186/s12870-015-0478-5) contains supplementary material, which is available to authorized users.

## Background

Sorbitol dehydrogenase (SDH, EC 1.1.1.14) is commonly found in all kinds of life forms, including animals [1-4], yeasts [5], bacteria [6] and plants [7-13]. It represents the early divergence within the NAD (H)-dependent medium-chain dehydrogenase/reductase (MDR) superfamily (with a typical ~350-residue subunit), sharing a distant homology with alcohol dehydrogenase (ADH, EC 1.1.1.1) [14-17]. SDH catalyses the reversible oxidation of a range of related sugar alcohols into their corresponding ketoses [7,13,18-21], preferring polyols with a d-*cis*-2,4-dihydroxyl (2S,4R) configuration and a C1 hydroxyl group next to the oxidation site at C2, such as sorbitol, xylitol and ribitol (Additional file 1). It exhibits the highest activity on sorbitol while also being able to oxidize the other polyols at lower reaction rates [6,13,18,20]. The process of sorbitol oxidation by human SDH requires a catalytic zinc atom which is coordinated by the side chains of three amino acids (44C, 69H, 70E, numbering in human SDH) and one water molecular. NAD^+^ binds to the protein first, followed by sorbitol. The backbone of sorbitol stacks against the nicotinamide ring while the C1 and C2 oxygen atoms are coordinated to the zinc. The water molecule coordinating the zinc atom acts a general base and abstracts the proton of the C2 hydroxyl, which creates an electron flow to NAD^+^, leading to the oxidation of sorbitol at C2 and the final production of NADH [22].

Plant SDH is the key enzyme in the sorbitol metabolism pathway [7,13,20,21,23] and has been associated with resistance to abiotic stresses such as drought and salinity. SDH activity regulates the levels of polyols [13,23], which act as important osmolytes during drought stress and recovery processes [24]. In *Rosaceae* species sorbitol occurs as the major photosynthate and phloem transported carbohydrate [25]. In these plants, which include apple [26-31], pear [32,33] and loquat [34,35], SDH plays a crucial role in the oxidation of sorbitol and its translocation to sink tissues such as developing fruits and young leaves. Gene transcript level and enzyme activity remain high during fruit development and maturation, dropping gradually in later stages, and contributing to the sugar accumulation in the ripening fruits [27-30,34-36]. The role of sink strength regulation for SDH is of particular research interest given the economic importance of these fruit species. Additionally, SDH has been shown to be involved in the sugar metabolism process during seed germination of some herbaceous plants including soybean [37] and maize [8,38].

Despite efforts to understand the physiological role of SDH in plants, little attention has been paid toward the evolutionary history of the plant SDH gene family. The distribution of the SDH genes in higher plants appears to be species-dependant. In particular, 9 paralogous SDH genes have been reported in apple [27] and 5 in Japanese pear [39]. In contrast, other plant genomes such as *A. thaliana* [23], tomato [11] and strawberry [12] contain only one SDH gene. Recent studies have indicated that there are two groups of SDH present in some *Rosaceae* plants. Park et al. [10] isolated four SDH isoforms (MdSDH1-4) from Fuji apple and found that MdSDH2-4 could be clearly distinguished from MdSDH1 based on the deduced amino acid sequence, showing 69–71% identity with MdSDH1 and 90–92% identity with each other. In addition, MdSDH2-4 were expressed only in sink tissues such as young leaves, stems, roots and maturing fruits while MdSDH1 was highly expressed in both sink and source organs [10]. Nosarzewski et al. [27] identified nine SDHs (SDH1-9) from the Borkh apple genome and showed that all isoforms except SDH1 (71–73% identity with SDH2-9) were highly homologous with an identity of 91–97%. Similar observations have been made with the SDH isoforms (PpySDH1-5) identified in pear whereby PpySDH5 differed from PpySDH1-4 at both the primary structure level and the gene transcriptional level [39]. Preliminary phylogenetic analyses have classified these homologous SDHs into two groups based on primary protein structures [10,29,33,40]. However, these studies focused on only one or just a few related *Rosaceae* species. No comprehensive phylogenetic analysis has been performed on SDH across a broad range of angiosperm species.

Gene duplication is widespread in plant genomes. Functional divergence after gene duplication is the major mechanism by which genes with novel function evolve; this phenomenon plays a key role in the evolution of phenotypic diversity [41-44]. The current understanding of gene evolution via duplication suggests that duplicated genes could arise through different mechanisms including unequal crossing over (resulting in tandem duplication), retrotransposition, segmental duplication and chromosomal (or whole genome) duplication [42,45]. Most duplicated genes are lost due to the accumulation of mutations that render them non-functional (pseudogenization) [42]. However, they can be retained under certain circumstances whereby the acquisition of beneficial mutations leads to novel function (neo-functionalization), which requires positive natural selection, or through adoption of part of the functions of the ancestral gene (sub-functionalization), which could occur by expression divergence or functional specialization of protein [41,42,46,47]. The latter usually involves a shift in the enzyme substrate specificity.

Protein structural analyses have shown that the LIDH of *V. vinifera*, which catalyses the inter-conversion of L-idonate and 5-keto-D-gluconate (5KGA) in the tartaric acid (TA) synthesis pathway [48], is highly homologous to plant SDHs, sharing ~77% amino acid sequence similarity with SDH from tomato (Gene ID: 778312) and *A. thaliana* (Gene ID: AT5G51970) [48]. The 366 amino acid LIDH (UniProt ID: Q1PSI9) contains an N-terminal GroES-like fold and a C-terminal Rossmann fold [48], characteristics of the ADH family [49], which has a distant homology to SDH [14-17]. However, unlike other plant SDHs, LIDH displays principal activity against L-idonate and has a low reaction rate with sorbitol [48]. The unique substrate specificity of LIDH was suggested to be due to small changes in amino acid sequence encoded by paralogous genes [48].

In this study, a comprehensive phylogenetic analysis of angiosperm SDHs was conducted using currently available genomic data. A computational approach was employed to characterise the natural selection pressure on plant SDH. The protein structures of the SDH homologues in *V. vinifera* were modelled based on human SDH (PDB:1PL8) to identify the putative active site residues of plant SDHs. Transcription and co-expression data of SDH genes were also extracted from recent publicly available microarray and co-expression databases and analysed. New insights into the evolution history of the plant SDH family and the evolutionary origin of *V. vinifera* LIDH will be discussed.

## Results and discussion

### Identification of sorbitol dehydrogenase (SDH) homologous genes in higher plants

**Figure 1:**
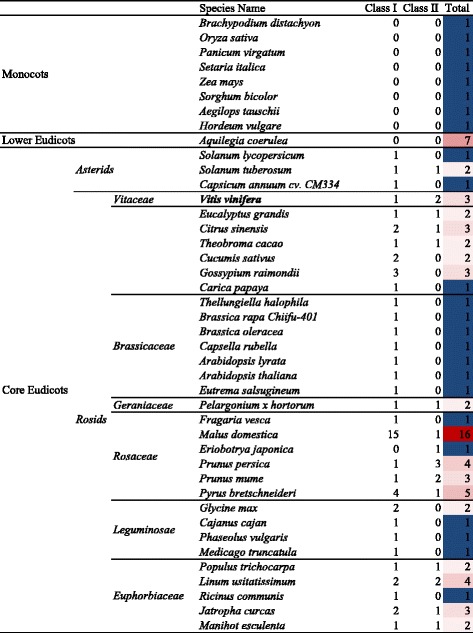
Distribution of SDH homologous genes in higher plants. Closely related species were specified accordingly. The gene abundance heat map was based on the total copy number of SDH genes in each species. SDHs of *P. bretschneideri* [39] and *E. japonica* (loquat) [35] were obtained from literature; additional SDHs may be identified in these two species when complete genome information becomes available. The classification of SDH Class I and SDH Class II was based on the phylogenetic analysis carried out in the present study.

A database homology search identified 92 SDH homologous genes from 42 species (Figure 1; See Additional file 2: Table S1 for identified gene IDs and Additional file 3 for gene sequences in corresponding species). At least one putative SDH gene was present in each plant genome studied, consistent with previous studies [17] that suggested the ubiquity of SDH and its functional importance across all life forms. However, the distribution of SDH homologous genes varied dramatically across species. Monocot species (n = 8) uniformly presented a single SDH gene, and this same observation was made with *Brassicaceae* plants (n = 7) from the Eudicot group. It was recently reported that there are 2 SDH genes in both rice (monocot) and *A.thaliana* (*Brassicaceae*) [50], however, in both cases these SDH genes were found to be alternative transcripts of a single gene. All except one species from the *Asterid* clade and the *Leguminosae* family had one SDH gene, the exceptions being *Solanum tuberosum* (potato) and *Glycine max* (soybean), respectively, which both had two copies. By contrast, numerous copies of SDH genes were found in *Rosaceae* species, which employ sorbitol as the major transported carbohydrate [25]. *Malus × domestica* (apple) contained 16 putative SDH genes, the highest number among all species investigated. A previous study [50] identified 17 SDH genes in the apple genome, however, the extra putative SDH (MDP0000506359) was only a partial gene (177 residues) and was excluded from the present study. In addition to apple, other *Rosaceae* species such as *Prunus persica* (peach), *Prunus mume* (Chinese plum), *Eriobotrya japonica* (loquat) and *Pyrus bretschneideri* (pear) had 4, 3, 1 and 5 putative SDH genes respectively. It should be noted that the information of SDH numbers in loquat [35] and pear [39] was retrieved from earlier reports, and that more SDH genes may be found when complete genome data for these species become available. Although *Fragaria vesca* (strawberry) belongs to the *Rosaceae* family, only one SDH gene was present in this species. Unlike other *Rosaceae* fruit species, *F. vesca* utilizes sucrose instead of sorbitol as the main translocated carbohydrate [51]. According to a recent development in the evolution by duplication theory, a proper gene dosage should be kept to maintain a stoichiometric balance in macromolecular complexes such as functional proteins, thereby ensuring the normal functioning of a particular biological process [41,52]. Transportation and assimilation of sorbitol is a *Rosaceae*-specific metabolism. The retention of highly duplicated SDH genes in *Rosaceae* species suggests that a higher dosage of SDH transcription or enzyme activity is needed to facilitate sorbitol metabolism in these species.

Three putative SDH genes were identified in the *V. vinifera* genome. One (GSVIVT01010646001) corresponded to the previously characterized LIDH (Uniprot No. Q1PSI9) [48] while the other two shared 99% (GSVIVT01010644001) and 77% (GSVIVT01010642001) amino acid sequence identity with *V. vinifera* LIDH (Additional file 2: Table S4). Other important crops such as *C. sinensis* (orange), *Theobroma cacao* (cocoa)*,* and *Pelargonium hortorum* (a geranium species) had 3, 2 and 2 SDH genes respectively. *P. hortorum* and *S. tuberosum* are of particular interest in this study because they have also been shown to accumulate significant levels of TA, like *V. vinifera* [53,54]. Another species that should be noted is *Aquilegia coerulea* (a flower native to the Rocky Mountains), which belongs to the Eudicot family but has been recognized as an evolutionary intermediate [55] between monocot and core Eudicot plants, and contained 7 SDH paralogues.

### Phylogenetic analysis of plant sorbitol dehydrogenase families

**Figure 2:**
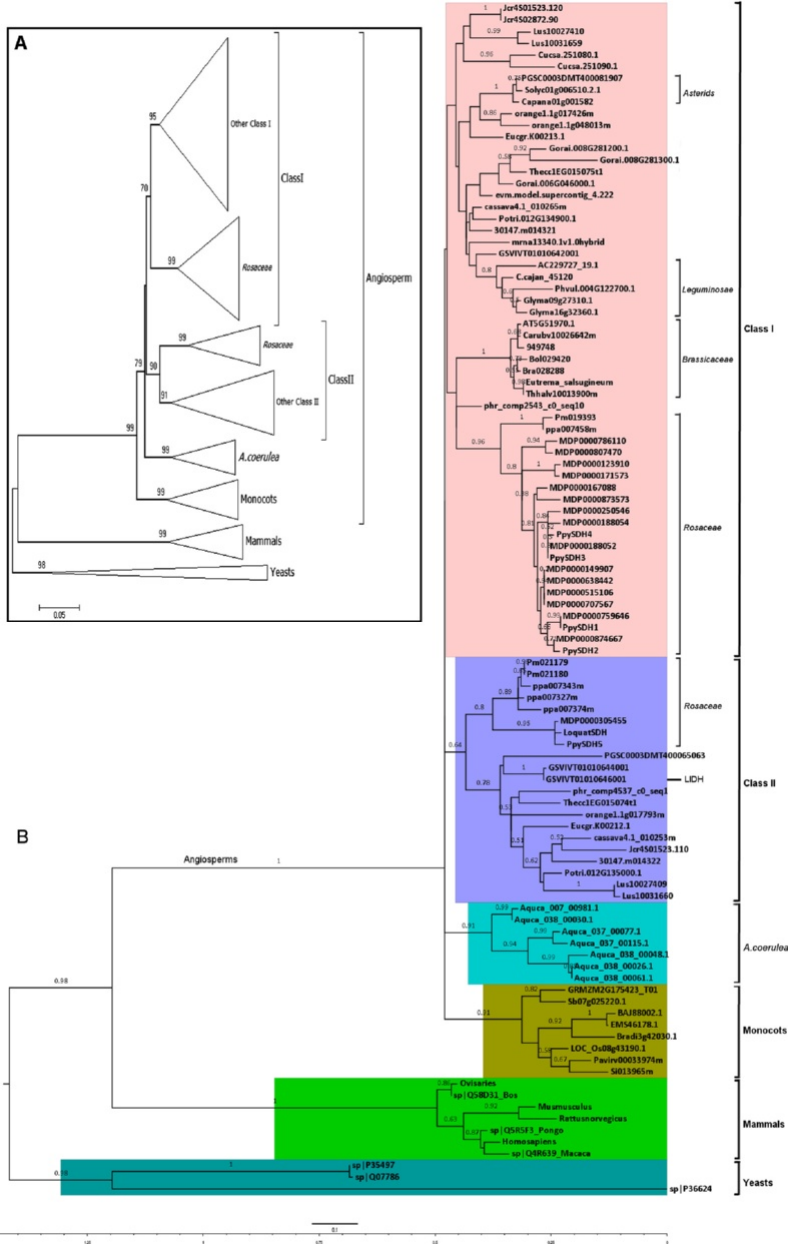
Phylogenetic tree showing the evolutionary history of the angiosperm SDH family. **A**: A simplified schematic phylogeny of the SDH family inferred by MEGA 6.0 [97] software using the Neighbour Joining method. Values (as percentage, cutoff value 50) of Internal branch test (1000 replicates) supports are indicated above the corresponding branches. **B**: The Maximum Likelihood phylogeny of the SDH family developed by MEGA 6.0 [97] software using the selected best-fitting substitution model JTT + G [99]. 1000 times Bootstraping supports (cut off at 0.5) are displayed above corresponding branch. Closely related species are annotated accordingly. The *V. vinifera* LIDH (GSVIVT01010646001) is also marked.

To determine the evolutionary history of plant SDH family and the phylogenetic relationship between LIDH and SDH, a phylogeny of the SDH family was reconstructed. Consistent results were obtained using both Neighbour Joining (Figure 2A; Additional file 4) and Maximum Likelihood (Figure 2B) methods. As can be seen in the Maximum Likelihood tree (Figure 2B), the target proteins divided at the basal nodes into three major clusters, corresponding to the three life kingdoms: fungi, animal and plant (Bootstrap supports at 0.98, 1 and 1 respectively). The overall topology of the plant SDH clade was in agreement with the Phytozome species tree (http://www.phytozome.net/), indicating that the phylogeny results were reliable. Specifically, monocot plants (n = 8) formed a single clade with strong support (0.91), corresponding to the early split between monocot and dicot lineages. *A. coerulea* SDHs separated into a single group (0.91) which positioned itself between monocot and core Eudicot plants. The *Aquilegia* genus belongs to the Eudicot order *Ranunculales* which has been established as a sister clade to the rest of the core Eudicot [56-58] and agrees with the present phylogenetic analysis.

The core Eudicot SDHs split into two distinct lineages in the Maximum Likelihood tree (Figure 2B). The first lineage (classified as Class I) covered all core Eudicot species included in this study while the second (Class II) had a narrower coverage and was less expanded compared to SDH Class I. The divergence of core Eudicot SDHs into two lineages was in agreement with previous reports that SDHs from some *Rosaceae* species could be separated into two groups [10,29,33]. All *Rosaceae* plants (n = 5) investigated in this study except *F. vesca* (strawberry) had multiple copies of SDH genes that covered both SDH Class I and SDH Class II. However, within these species, the distribution of SDHs among the two SDH classes varied greatly. In particular, 15 out of the 16 SDHs from *M. domestica* and 4 out of the 5 SDHs from *P. bretschneideri* fell into SDH Class I while 3 out of the 4 SDHs from *P. persica* and 2 out of the 3 SDHs from *P. mume* belonged to SDH Class II. Other species retaining two classes of SDHs included *S. tuberosum*, *V. vinifera*, *Eucalyptus grandis*, *C. sinensis*, *T. cacao*, *P. hortorum*, *Populus trichocarpa*, *Linum usitatissimum*, *Jatropha curcas* and *Manihot esculenta*, from different orders or families. In contrast, *Brassicaceae* plants (n = 7), *Leguminosae* plants (n = 4) and *Asterid* plants (n = 2) except *S. tuberosum* contained either a single SDH or two SDHs that could only be classified into SDH Class I. Within both SDH Class I and Class II clades, *Rosaceae* SDHs (except *F. vesca*) formed separate phylogeny groups (Figure 2B), implying divergent molecular characteristics for SDHs from this family. Most recent phylogenetic analyses [59,60] have placed *Vitaceae* as a sister clade to the *Rosid* plants in the core Eudicot group. The presence of two classes of SDHs in both *V. vinifera* and *S. tuberosum* (*Asterid*s) indicated that the divergence between SDH Class I and Class II occurred before the species radiation of the core Eudicot plants. Moreover, although 7 SDH genes were retained in the genome of the evolutionarily intermediate species *A. coerulea*, none of them could be classified into SDH Class I or SDH Class II. Taken together, our results suggested that SDH Class I and Class II might have diverged during the common ancestor of core Eudicot plants but after the branching of the basal Eudicots such as *Ranunculales.* This corresponds to a period of about 125Mya ~ 115Mya [55,58].

In the Maximum Likelihood tree, the Class II clade was well-supported and separated from Class I with longer branch length in general (Figure 2B), suggesting a higher level of amino acid substitution within this clade. In addition, the topology of the Class II clade (except the *Rosaceae* group) was in good agreement with the species tree at Phytozome (http://www.phytozome.net/search.php), with *S. tuberosum* (*Asterids*) diverging first followed by *V. vinifera* and the rest of the rosid species. This indicates that the Class II SDHs have evolved vertically within respective species, which lends further support to the suggestion above that SDH Class I and Class II have existed during the common ancestry of core Eudicot plants. The backbone topology of the more inclusive Class I clade in the Maximum Likelihood tree was weakly supported (Bootstrap support under 0.5; Figure 2B), in contrast with the strong clustering support for this clade in the Neighbour Joining tree (Figure 2A; Additional file 4). The weak bootstrap support for the topology of SDH Class I may have resulted from a lack of amino acid substitution in this clade, as reflected by the short branch length (Figure 2B). The calculation of evolutionary distances for plant SDHs revealed a pair-wise distance under 0.3 in general (Additional file 2: Table S2), sequence alignment showed that Class I SDHs tend to be more conserved (average sequence pair-wise identity 83.4%; Table 1) than Class II (79%; Table 1), which means less amino acid substitution within the Class I clade. These results are consistent with the strong clustering support for the major sub-clades of the Class I branch in the Neighbour Joining tree (Figure 2A; Additional file 4).

**Table 1:** **Amino acid sequence identity between different SDH groups**

**Identity**	**Class I**	**Class II**	***A. coerulea***	**Monocot**	**Mammal**	**Yeast**
**Class I**	83.4 (71-99.7)	75.2 (67-83)	78.5 (71-86)	77.5 (71-83)	48.0 (44-50)	40.9 (38-43)
**Class II**		79.0 (71-99)	73.2 (68-80)	71.0 (67-74)	46.4 (43-49)	39.3 (37-42)
***A. coerulea***			86.7 (83-99.7)	75.7 (72-79)	48.0 (47-50)	41.4 (40-43)
**Monocot**				88.4 (86-93)	47.4 (46-49)	41.5 (40-45)
**Mammal**					87.8 (82-99.8)	42.3 (39-44)
**Yeast**						65.5 (48-99.7)

SDH sequences were divided into six groups (Class I, Class II, *A. coerulea*, Monocot, Mammal and Yeast SDHs) according to the phylogenetic analysis carried out in the present study (Figure 2). The amino acid sequence identity (as percentage) was obtained using all-vs-all BLAST tool. The average pair-wise identity between each group is presented, followed by the identity range (in bracket).

In contrast to the ubiquity of Class I SDHs, the absence of Class II SDHs in some species may be due to gene loss after duplication, a common mechanism in gene evolution via duplication [42,61]. This also indicated that SDH Class II members may not be essential for the normal growth of plants, suggesting a divergent function for this class of SDH genes. Interestingly, the previously characterized *V. vinifera* LIDH (GSVIVT01010646001) [48] was grouped into SDH Class II, providing direct support that in at least one case SDH Class II may have acquired a novel function, in this instance its involvement in the synthesis of TA. While the identity of additional functions for Class II SDHs in other species is unknown, support for a role of some Class II SDHs in TA metabolism may be proposed. Only a few plant families, including *Vitaceae*, *Geraniaceae* and *Leguminosae* have been shown to accumulate significant levels of TA [54] and the present results showed that Class II SDHs were present in both *Vitaceae* and *Geraniaceae*. The absence of Class II SDHs in *Leguminosae* plants could be explained by the fact that the synthesis of TA in *Leguminosae* proceeds via a different pathway, which bypasses the interconversion of L-idonate and 5KGA (catalysed by LIDH) [62]. Recent studies have revealed that potato [53], citrus fruits [63] and pear [64,65] (all containing Class II SDHs) also produce TA, although to a lesser degree than *V. vinifera*. This is consistent with the potential correlation between Class II SDHs and TA synthesis. However, it has also been reported that TA is absent or found only in trace amount in apple [66], and no information is available about the occurrence of TA in peach even though three copies of Class II SDH genes were identified in this species (Figure 1). It is possible that Class II SDHs have evolved varied functions to meet the different environmental challenges faced by respective plants. In this context, it would also be valuable for future work to investigate the in-planta function of SDH and the occurrence of TA in the evolutionarily intermediate plant *A.coerulea*, for which 7 SDH paralogues were identified.

### Sequence alignment and protein subdomain analysis

**Figure 3:**
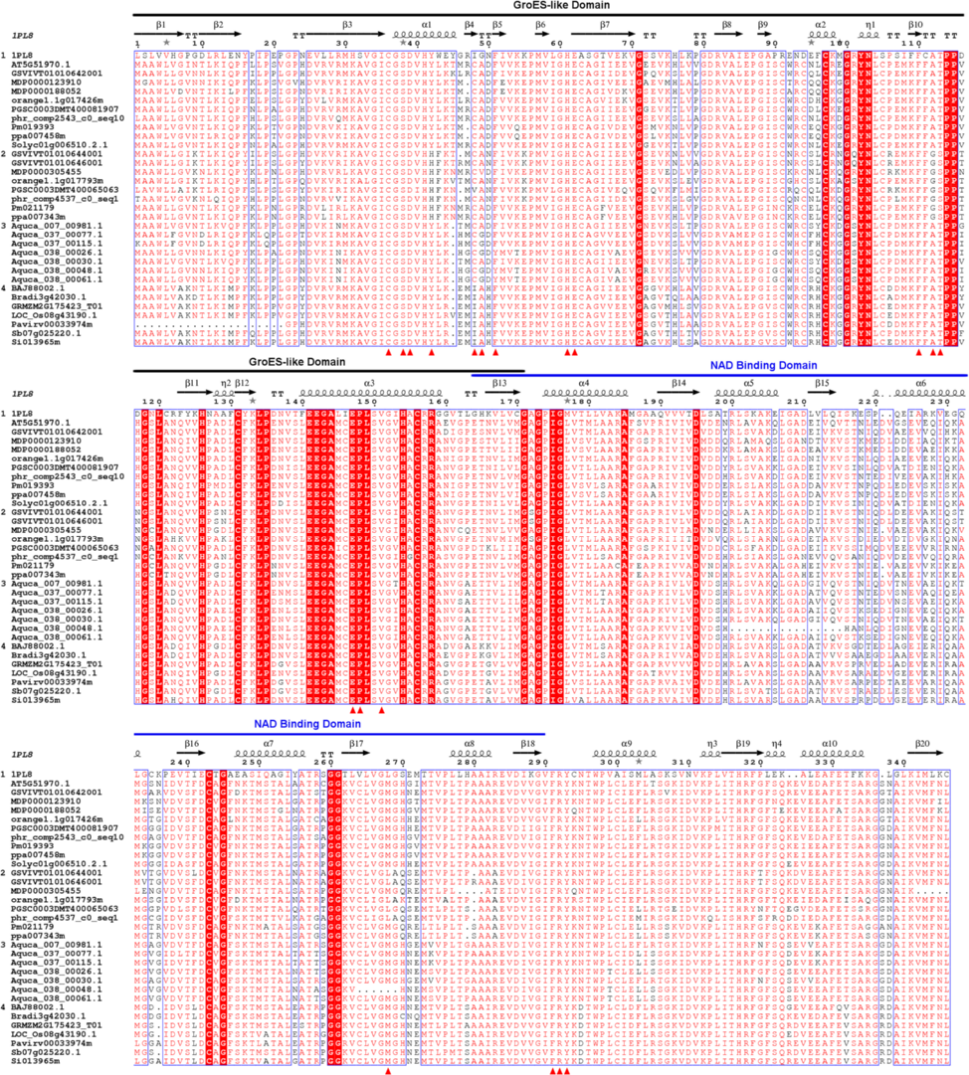
Multiple sequence alignment of plant SDH family. ESPript output was obtained with the sequence alignment of plant SDHs and human SDH. Secondary structures were inferred using human SDH (PDB: 1PL8) as a template, with springs representing helices and arrows representing beta-strands. Sequences were grouped into 1 (1PL8 and core Eudicot SDH Class I), 2 (core Eudicot SDH Class II), 3 (*A.coerlea* SDH) and 4 (monocot SDH). Amino acid site numbering above the alignment is according to LIDH (Q1PSI9) without the first 20 amino acids. Adjacent similarity amino acid sites were boxed in blue frame. Similarity calculations were based on the complete SDH alignments but only partial sequences for SDH Class I and SDH Class II were displayed. The active site residues identified in this study are marked with red triangles. Conserved domains are indicated above the alignment.

Sequence alignment and protein subdomain analyses were performed to investigate the molecular characteristics of plant SDHs. Results showed that plant SDHs shared an overall identity above 67% (Table 1), while having ca 48% and ca 41% identities with mammal and yeast SDHs respectively (Additional file 2: Table S4). Plant SDHs were clustered into four groups in the present phylogenetic analysis: monocot SDH, *A. coerulea* SDH, core Eudicot SDH Class I and SDH Class II. Protein BLAST results showed that Class I and Class II SDHs within the same species generally had an inter-class identity of around 70% and an intra-class identity above 90% (Additional file 2: Table S4). When compared with monocot and *A. coerulea* SDHs, Class I SDHs always demonstrated a significantly higher similarity than Class II SDHs (77.5% vs 71.0% and 78.5% vs 73.2% respectively; Table 1), suggesting that core Eudicot Class I SDHs have a closer distance to monocot and *A. coerulea* SDHs and that SDH Class II may have diverged from SDH Class I. In addition, Class I SDHs tend to be more homologous than Class II SDHs (83.4% vs 79.0%; Table 1). No significant difference between the two SDH classes was observed when compared to mammal or yeast SDHs (48.0% vs 46.4% and 40.9% vs 39.3% respectively; Table 1). Protein functional domain prediction identified two functional domains for plant SDHs: an N-terminal GroES-like fold and a C-terminal Rossmann fold (Figure 3; See Additional file 5 for the complete sequence alignment). Secondary structure analysis showed that these two domains tended to be highly conserved among all plant SDHs, and amino acid substitutions mainly occurred at boundary regions linking secondary structural elements such as alpha-helices and beta-sheets (Figure 3).

### Gene duplication pattern characterization and synteny analysis

To characterise the expansion patterns of plant SDH gene family, nine species that were from different families and contained both classes of SDHs were selected for gene duplication and synteny analyses (*C. sinensis*, *E. grandis*, *P. mume*, *P. persica*, *Populus trichocarpa*, *M. domestica*, *S. tuberosum*, *T. cacao* and *V. vinifera*). As shown in Table 2 (See Additional file 6 for the original output data), tandem duplication contributed the most to the expansion of the core Eudicot SDH family, followed by WGD/Segmental duplication. Dispersed SDHs (MDP0000305455, MDP0000759646 and PGSC0003DMC400055323) and a single proximal SDH (MDP0000188054) were identified only in *M. domestica* and *S. tuberosum*. Based on phylogenetic classification in the present study, Class I and Class II SDH genes from *E. grandis*, *P. trichocarpa*, *T. cacao* and *V. vinifera* are located in a tandem manner in their corresponding chromosomes, which provides strong support that SDH Class I and SDH Class II are tandem duplications. A similar pattern was observed with *C. sinensis* whereby Cs9g16660.1 (SDH Class II) is separated by a single-gene insertion with the two Class I SDH genes (Cs9g16680.1, Cs9g16690.1; data not shown). This may be caused by gene insertion after tandem duplication. Class I and Class II SDH genes in the three *Rosaceae* species (*M. domestica*, *P. mume*, *P. persica*) and in *S. tuberosum* are separated either on the one chromosome or on separate chromosomes altogether, indicating a divergent evolutionary history for SDH genes in the *Rosaceae* family and in *S. tuberosum* compared to other plants. SDH genes on chromosome 1 (md1) and chromosome 7 (md7) in *M. domestica* were highly duplicated by tandem duplication (Table 2), in contrast to the other *Rosaceae* species (*P. mume*, *P. persica*). Notably, the Class I SDH gene from *S. tuberosum* (PGSC0003DMC400055323) and the Class II SDH gene from *M. domestica* (MDP0000305455) were identified as dispersed duplicates, which may underpin the divergent sorbitol metabolism profiles across these species.

**Table 2:** **Gene duplication patterns of plant SDH**

**Species**	**Chromosome ID**	**SDH gene ID**	**SDH class**	**Duplication pattern**	**Start position**	**End position**
*C. sinensis*	cs9	Cs9g16680.1 (orange1.1g017426m)	I	Tandem	16143063	16147624
	cs9	Cs9g16690.1 (orange1.1g048013m)	I	Tandem	16150122	16154404
	cs9	Cs9g16660.1 (orange1.1g017793m)	II	WGD or Sgm	16135216	16138066
*E. grandis*	eg11	Eucgr. K00213.1	I	Tandem	2624187	2627945
	eg11	Eucgr.K00212.1	II	Tandem	2615486	2618589
*M. domestica*	md1	MDP0000786110	I	Tandem	25191824	25193641
	md1	MDP0000873573	I	Tandem	25182502	25183812
	md1	MDP0000707567	I	Tandem	25180931	25182241
	md1	MDP0000515106	I	Tandem	25177288	25178612
	md1	MDP0000250546	I	Tandem	25173127	25174375
	md1	MDP0000874667	I	Tandem	25157544	25158783
	md1	MDP0000638442	I	WGD or Sgm	25149134	25150444
	md1	MDP0000123910	I	WGD or Sgm	25087036	25088743
	md1	MDP0000305455	II	Dispersed	14150327	14159200
	md7	MDP0000188052	I	Tandem	23301490	23302735
	md7	MDP0000171573	I	WGD or Sgm	23281847	23283529
	md7	MDP0000188054	I	Proximal	23310942	23312187
	md7	MDP0000167088	I	Tandem	23405354	23406795
	md7	MDP0000807470	I	WGD or Sgm	23390960	23392683
	md14	MDP0000759646	I	Dispersed	24043122	24044360
*P. mume*	Pm5	Pm019393	I	WGD or Sgm	23673441	23675177
	Pm6	Pm021180	II	Tandem	7217228	7219256
	Pm6	Pm021179	II	Tandem	7217228	7225304
*P. persica*	pp2	ppa007458m|PACid:17644502	I	WGD or Sgm	24766424	24768515
	pp4	ppa007327m|PACid:17655491	II	WGD or Sgm	17729024	17731238
	pp8	ppa007343m|PACid:17644328	II	Tandem	15254677	15256888
	pp8	ppa007374m|PACid:17655656	II	Tandem	15249947	15251989
*P .trichocarpa*	pt12	POPTR_0012s13780	II	WGD or Sgm	13789342	13787442
	pt12	POPTR_0012s13790	I	WGD or Sgm	13790093	13792804
*S. tuberosum*	st01	PGSC0003DMC400055323	I	Dispersed	1594220	1598967
	st06	PGSC0003DMC400043871	II	WGD or Sgm	24156879	24158593
*T. cacao*	tc03	Tc03_g019280	I	WGD or Sgm	18300080	18303115
	tc03	Tc03_g019270	II	WGD or Sgm	18298897	18296706
*V. vinifera*	vv16	GSVIVT01010642001	I	WGD or Sgm	15653874	15651701
	vv16	GSVIVT01010646001	II	Tandem	15675560	15678887
	vv16	GSVIVT01010644001	II	Tandem	15666264	15664425

SDH gene duplication patterns were characterized by the *duplicate_gene_classifier* program in the MCScanX package. “WGD or Sgm” refers to Whole Genome Duplication or segmental duplication. “SDH Class” is defined according to the present phylogenetic analysis. Notably, MDP0000149907 from *M. domestica* could not be anchored in any chromosome and was therefore absent in this table.

**Figure 4:**
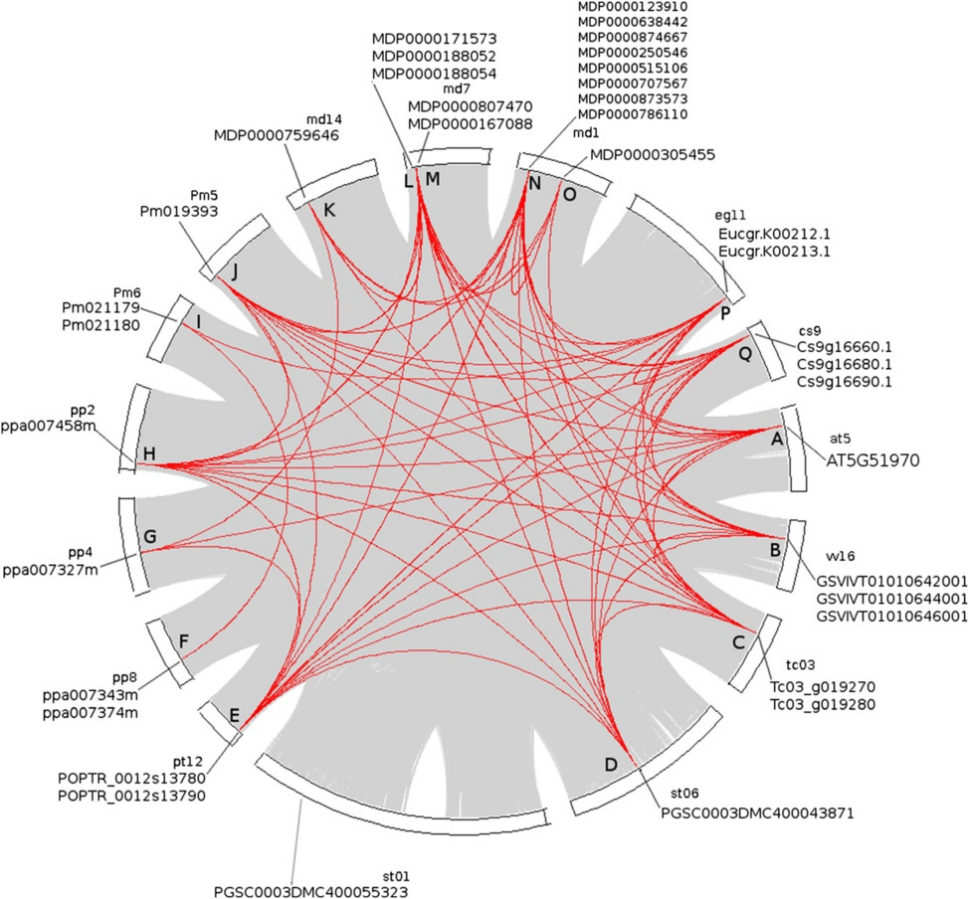
Identification of collinear gene pairs among plant SDH families. A circular plot of SDH gene family collinearity. Collinear SDH genes are linked by red curved lines. SDH genes located at each position in corresponding chromosomes are indicated. Family collinearity is shown in the genomic collinearity background. Only those chromosomes containing SDH genes are included.

To investigate the conservation of SDH genes across species, collinear SDH gene pairs were identified within and across species. SDH genes from the nine above-mentioned species were analysed. The single SDH gene (AT5G51970) from the model plant *A. thaliana* was also used as a reference for collinear block identification. As shown in Figure 4, all target plant genomes contained at least one SDH gene (corresponding to chromosome positions A, B, C, D, E, H, J, L, N, P and Q in Figure 4) with collinear SDH genes in all other nine species studied, indicating a conserved collinear SDH block. SDH genes at gene positions F, G, I, K and O, concerning only the *Rosaceae* species investigated, were collinear with SDH genes in only some of the species included in the present analysis. In particular, position F at chromosome 8 (pp8) of *P. persica* paired only with position I at chromosome 6 (Pm6) of *P. mume*. While position F was found collinear only with position I, position I had another collinear region at position O from *E. grandis*. Position G at chromosome 4 (pp4) of *P. persica* was only paired with positions A, E and K from *A. thaliana*, *P. trichocarpa* and *M. domestica* respectively. Some collinear SDH gene pairs, such as F-I, G-K and K-O, were restricted to *Rosaceae* species only, reflecting genetic features shared only by these plants. Notably, intra-species collinear SDH pairs were identified only within *M. domestica* but not in *P. mume*, *P. persica* and *S. tuberosum* although all of these species have SDH genes located on multiple chromosomes (Figure 4; See Additional file 2: Table S5 for identified collinear SDH gene pairs). This observation could be explained by the fact that the apple genome underwent a recent (>50Mya) WGD, which doubled the chromosome number from nine to 17 in the *Pyreae* [50] while most other *Rosaceae* plants have a haploid chromosome number of 7, 8 or 9. *S. tuberosum* was unique among the species investigated in that it had a Class II SDH gene (PGSC0003DMC400043871) but no Class I SDH gene preserved in the collinear region (Figure 4). The Class I SDH gene (PGSC0003DMC400055323), which was identified as a dispersed duplication (Table 2), was the only SDH gene for which no collinear gene was identified in the present analysis. Since the Class II SDH homologue (LIDH) in *V. vinifera* has been shown to be involved in TA synthesis [48], it would be of great interest to investigate the potential role of SDHs in *S. tuberosum*, which has also been shown to accumulate a significant amount of TA [53]. Noteworthy, *S. lycopersicum*, another species from the *Solanale* order, accumulates no TA [67] and contains only a single SDH, which belongs to Class I (Figure 2B).

### Natural selection analysis

Assessment of synonymous and non-synonymous substitution ratios is important to understand molecular evolution at the amino acid level [68,69]. To examine the intensity of natural selection acting on the specific clade, the ratio (w) of non-synonymous substitution to synonymous substitution in the developed plant SDH phylogeny was investigated, whereby w<1, w=1 and w>1 indicated purifying selection, neutral evolution and positive selection respectively. Based on our phylogeny results, four branches (“monocot SDH”, “*A. coerulea* SDH”, “core Eudicot SDH Class I” and “core Eudicot SDH Class II”) were specified for w assessments (w [mono], w [Aer], w [sdhC1] and w [sdhC2] respectively). Firstly, the branch-specific likelihood model [70] was applied to the SDH data. As can be seen in Table 3, Likelihood-ratio tests (LRT) showed that the two-ratio model and the four-ratio model fit the dataset significantly better (2∆*l* = 12.6 with p = 0.0004, df = 1 and 2∆*l* = 13.2 with p = 0.0042, df = 3 respectively) than the one-ratio model. In contrast, the three-ratio model assumption lacked statistical support (2∆*l* = 0.2 with p = 0.9048, df = 2). Given that the two-ratio and four-ratio models assume unequal w ratios for the Class I and Class II branches while the three-ratio model specifies w(sdhC1)=w(sdhC2) (Table 3), the above calculation suggested that the w ratio for the core Eudicot SDH Class II was significantly different from that of Class I. Moreover, the four-ratio model, which assumes unequal w ratios for the monocot, *A.coerulea* and Class I branches (Table 3), was not significantly better (2∆*l* = 0.6 with p = 0.7408, df = 2) than the two-ratio model (assuming uniform ratio for these branches; Table 3). This indicated that the w ratios for monocot, *A. coerulea* and core Eudicot Class I branches had no significant difference. Notably, all branch-specific models tested demonstrated a low w value for the monocot, *A. coerulea* and Class I branches (w[mono]=w[Aer]=w[sdhC1]=0.10415 with the two-ratio model and w[mono]=0.10428, w[Aer]=0.09731, w[sdhC1]=0.0001with the four-ratio model), suggesting that plant SDHs have been under strong purifying selection. This agrees well with the suggestion that functional proteins are usually under strong structural and functional constraints [71]. It should be noted that w[sdhC2] were infinite in both multi-ratio models (w[sdhC2]=859 and 999 respectively). This is because an extremely low level of synonymous substitution or no synonymous substitution was detected in the SDH Class II clade. On the other hand, the number of non-synonymous substitutions in the core SDH Class II clade was estimated to be 12.7 and 12.8 respectively for the two-ratio model and the four-ratio model. In contrast, only 0.4 non-synonymous substitution was detected for the SDH Class I clade with the two-ratio model (Additional file 7: branch-specific-two-ratio-output) and no non-synonymous substitution was detected with the four-ratio model (Additional file 7: branch-specific-four-ratio-output). These results provided clear evidence that positive selection had occurred in the lineage leading to core Eudicot SDH Class II. To test whether w[sdhC2] is significantly higher than 1, the log likelihood value (Table 3; Additional file 7: branch-specific-two-ratio-null-output) was calculated for the two-ratio model with w[sdhC2]=1 fixed. Results showed that this model was not significantly worse than the two-ratio model without the “w[sdhC2]=1” constraint (2∆*l* = 0.6 with p = 0.4386, df = 1), suggesting that w[sdhC2] was not significantly greater than 1 at the 5% significance level. This leads to the hypothesis that positive selection in SDH Class II might have only affected particular amino acid residues in the protein sequence, which is possible for a functional protein under strong structural and functional constraints [72]. To test this, Site-specific likelihood analysis was performed on the same data, which assumes variable selection pressures among amino acid sites but no variation among branches in the phylogeny. Results (Table 3: model M2) showed that the selection model (M2) fitted the dataset significantly better (2∆*l* = 994.8 with p = 0.0001, df = 2) than the one-ratio model but was not better (2∆*l* = 0 with p = 1, df = 1) than the neutral model (M1). These results indicated a significant variation of selection pressure among amino acid sites of plant SDH. However, the Selection model failed to detect any positively selected amino acid site at a significant level (Table 3; Additional file 7: site-specific-output), which suggested that no positively selected amino acid site could be identified across all branches. Therefore, we speculate that the positive selection might have only acted on a few amino acid sites in the core Eudicot SDH Class II clade.

**Table 3:** **Natural selection tests of plant SDH**

**Model**	**np**	***l*** **= ln L**	**Estimates of parameters**	**Positively selected sites**
**M0: one-ratio**				
w(mono)=w (Aer)=w(sdhC1)=w(sdhC2)	1	-30147.4	w(mono)=w(Aer)=w(sdhC1)=w(sdhC2)=0.10492	Not Allowed (NA)
**Branch-specific models**				
w(mono)=w(Aer)=w(sdhC1)≠w(sdhC2) (two ratios)	2	-30141.1	w(mono)=w(Aer)=w(sdhC1)=0.10415, w(sdhC2)=859.33956	NA
w(mono)≠w(Aer)≠w(sdhC1)=w(sdhC2) (three ratios)	3	-30147.3	w(mono)=0.10510, w(Aer)=0.10821, w(sdhC1)=w(sdhC2)=0.06935	NA
w(mono)≠w(Aer)≠w(sdhC1)≠w(sdhC2) (four ratios)	4	-30140.8	w(mono)=0.10428, w(Aer)=0.09731, w(sdhC1)=0.0001, w(sdhC2)=999	NA
w(mono)=w(Aer)=w(sdhC1)≠w(sdhC2) (two ratios with w(sdhC2) fixed to 1)	1	-30141.4	w(mono)=w(Aer)=w(sdhC1)=0.10424 (w(sdhC2)=1)	NA
**Site-specific models**				
M1:Neutral (2 site classes)	2	-29650.0	p0=0.87775 (p1=1-p0=0.12225); w0=0.07628 (w1=1)	NA
M2:Selection (3 site classes)	3	-29650.0	p0=0.87775, p1=0.07499 (p2=1-p0-p1=0.04726); w0=0.07628 (w1=1), w2=1	None
**Branch-site models **(SDH Class II as foreground lineage)				
Model A Null (4 site classes)	3	-29643.2	p0=0.33951, p1=0.04783 (p2+p3=0.61266); w0=0.07544	NA
Model A (4 site classes)	4	-29640.9	p0=0.82864, p1=0.11666 (p2+p3=0.0547), w0=0.07544 (w1=1), w2=132.6226	Sites for foreground lineage: 42H,43F,112G, 113S,116T, 270Q (p > 0.99);

All calculations were implemented using codeml at PAML4.7. Different models were specified according to the software instruction. “np” refers to the number of parameters, “*l* = (ln L)” refers to the log value of the likelihood. The estimated parameters w and p refer to the K_a_/K_s_ ratio and the percentage of the corresponding site classes respectively. In the one-ratio model M0 and the Branch-specific models, w(mono), w(Aer), w(sdhC1) and w(sdhC2) stand for the w ratios for the monocot, *A. coerulea*, SDH Class I and SDH Class II branches respectively. In the Site-specific models and the Branch-site models, w0, w1 and w2 represent the w ratios for the specific site classes in respective models (see the Methods section for more details). For the Branch-site models, the SDH Class II branch was specified as the foreground branch. Amino acid site numbering is according to LIDH (Uniprot No: Q1PSI9) without the first 20 amino acids.

In this context, a Branch-site model [73] that permits variable w ratios among both amino acid sites and branches was applied. Model A successfully identified the potential amino acid sites under positive selection in the SDH Class II branch (Table 3; Additional file 7: branch-site-modelA-output). Specifically, 42H, 43F, 112G, 113S, 116T and 270Q (numbering in LIDH (Q1PSI9) without the first 20 amino acids) were identified with Model A (Bayes Empirical Bayes analysis possibility >0.99; Additional file 7: branch-site-modelA-output). LRTs test showed that Model A fit the data significantly better (2∆*l* = 18.2 with p = 0.0001, df = 2) than the neutral model M1. The comparison (2∆*l* = 4.6 with p = 0.0320, df = 1) of Model A with its null hypothesis which assumes w2=1 (Additional file 7: branch-site-modelA-null-output) indicated that these amino acid sites had undergone positive selection in SDH Class II but not in the background branches. In addition, the Model A test demonstrated that 82.90% (model A: p0 = 0.82864; Table 3) of the amino acids of SDH were under strong purifying selection (model A: w0=0.07544; Table 3) and 11.7% were under neutral selection (model A: p1=0.11666, w1=1; Table 3) in all branches. No positive selection could be detected in the background branches (Additional file 7: branch-site-modelA-output). Taken together, these calculations demonstrated that plant SDHs were under strong purifying selection pressure and were highly conserved across all the plant species, and more importantly, that positive natural selection had occurred in the SDH Class II clade, affecting specific amino acids, namely 42H, 43F, 112G, 113S, 116T and 270Q.

### Ancestral sequence reconstruction and evolution rate analysis

To characterize the evolutionary rates for different groups of plant SDHs, ancestral amino acid sequences for the developed SDH phylogeny were reconstructed. Results (Additional file 8: ancestral-sequence-construction-output) showed that 9 potential amino acid substitutions (Y42H, L43F, A112G, T113S, V116T, Q228K, H270Q, N271S, R283A; numbering in LIDH (Q1PSI9) without the first 20 amino acids) occurred in the branch leading to SDH Class II from the common ancestor of core Eudicot SDH. This finding corresponded well with the natural selection analysis, whereby six out of the nine amino acid sites were identified to be under positive selection (42H, 43F, 112G, 113S, 116T and 270Q; Table 3). In contrast, no substitution was detected in the branch leading to core Eudicot SDH Class I (Additional file 8: ancestral-sequence-construction-output and interpreted-ancestral-sequences.fasta). Relative rate tests (RRT) [74] using monocot SDH as the out-group showed that core Eudicot SDH Class II evolved significantly faster than core Eudicot SDH Class I (Additional file 9: ClassI-vs-ClassII.txt), indicating a relaxed selection pressure on SDH Class II. In contrast, *A. coerulea* SDH and core Eudicot Class I SDH demonstrated no significant difference (Additional file 9: Aer-vs-ClassI.txt).

### Protein structure modelling analysis

**Figure 5:**
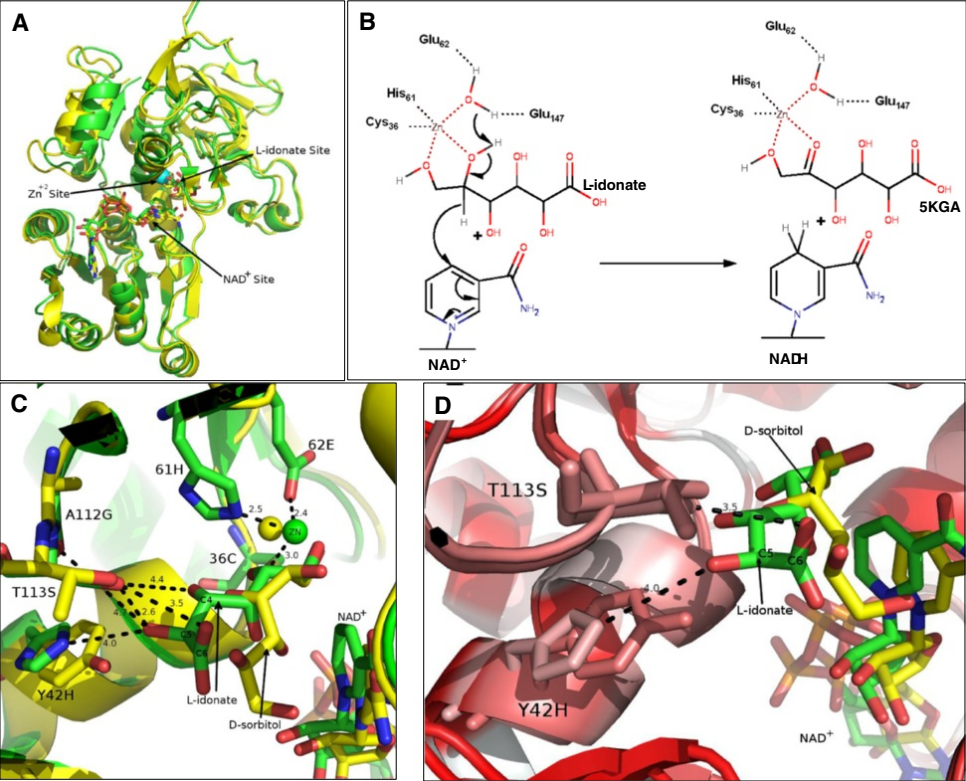
Homology models of Vv_LIDH and Vv_SDH and proposed reaction mechanisms. **A**. Structure superimposition of Vv_LIDH_idonate (green) and Vv_SDH_sorbitol (yellow) in Ribbon forms. **B**. The proposed reaction mechanism for Vv_LIDH on the oxidation of L-idonate into 5-keto-D-gluconate (5KGA). **C**. Superimposition of the active site residues of Vv_LIDH (green) and Vv_SDH (yellow). The distances (Å) between corresponding atoms are labelled. Target active site residues are shown in stick forms and labelled correspondingly. **D**. Hydrophobicity variance at Y42H between Vv_LIDH (green) and Vv_SDH (yellow) with red and white colours representing the highest hydrophobicity and the lowest hydrophobicity respectively. (All amino acid site numbering is according to LIDH (UniProt No: Q1PSI9) without the first 20 amino acids).

To deduce the reaction mechanism and identify the potential active sites of plant SDHs, protein structure models of *V. vinifera* Class I SDH (Vv_SDH, UniProt No: D7TMY3) and Class II SDH (Vv_LIDH, UniProt No: Q1PSI9) were created based on human SDH (PDB: 1PL8; 46 ~ 47% identity with Vv_SDH and Vv_LIDH). Ligands including zinc, NAD^+^, D-sorbitol and L-idonate were docked into the models (Additional file 10). Our models contain one zinc binding site, located in the active site. Some published SDH crystal structures (eg. PDB: 1E3J) contain a second, structural zinc-binding site distant from the active site catalytic zinc atom; this is not however a universal feature of these enzymes. No function has been correlated with the second, structural zinc-binding site. The sequence of our homology models does not support a second, structural zinc-binding site, as the necessary side chains required for zinc coordination are absent. A ribbons diagram of the overall structure of the homology models can be seen in Figure 5A, with Vv_SDH and Vv_LIDH adopting a typical dehydrogenase fold with an NAD^+^ binding site conforming to a Rossmann fold. The catalytic zinc ion in the active site was modelled coordinating to 36C, 61H and 62E (Figure 5C; numbering in LIDH (Q1PSI9) without the first 20 amino acids). All three of these residues together with 147E (corresponding to 155E in human SDH, mediating the water molecule coordinating the zinc atom [22]) are strictly conserved in plant SDHs (Figure 3). The 2′ and 3′ hydroxyls of the NAD^+^ ribose in our model were poised to 195D (203D in human SDH), potentially forming hydrogen bonds (Additional file 10: Asp195-NAD.png). The preservation of 195D instead of 195A at this amino acid site has been shown to be the structural basis for the selection of NAD (H) over NADP (H) as coenzyme [75]. This amino acid site is strictly conserved in all plant SDHs (Figure 3), implying that plant SDHs preferably utilize NAD (H). This suggestion is consistent with the lack of NADP-SDH activity for plant SDHs [7,10,11,13]. Previous characterizations of SDHs from Arabidopsis [13], tomato [11], apple [7,76] and pear [20] have suggested that plant SDHs exhibit highest activity for the oxidation of sorbitol, while also being able to oxidize other polyols such as xylitol and ribitol at lower reaction rates. However, the characterization of *V. vinifera* LIDH showed that this enzyme demonstrated the highest reaction rate on L-idonate but had a low reaction rate with sorbitol [48]. Upon docking of L-idonate, we found overall similar hydrogen bonding patterns with sorbitol as those proposed by Pauly et al. [22] and Yennawar et al. [77]. Earlier studies on enzyme substrate specificity also indicated that SDHs preferentially use substrates with a d-cis-2,4-dihydroxyl (2S,4R) configuration [6,13,18,20] (Additional file 1). L-idonate and D-sorbitol have the same molecular configuration from C1 to C4 and differ only at C5 (D and L chirality) and C6 (a hydroxyl group in sorbitol is replaced by a carboxyl group in L-idonic acid) (Additional file 1). Protein modelling analyses showed that L-idonate occupied a comparable position in the active site to sorbitol (Figure 5C). Therefore a similar reaction mechanism for L-idonate oxidation by *V. vinifera* LIDH is possible with D-sorbitol oxidation by human SDH [22]. The hydroxyl groups at C1 and C2 of L-idonate were modelled within interacting distance of the zinc atom in *V. vinifera* LIDH (Additional file 10: C1-C2-Zn.png), which may facilitate the proton transfer from C2 hydroxyl to NAD^+^, ultimately resulting in an oxidized C2 with ketone and the production of NADH (Figure 5B). Previous work suggested that the preferential binding of L-idonate over sorbitol seen in *V.vinifera* LIDH may be attributed to amino acid substitution at the catalytic sites between paralogous proteins [48]. As a result, the catalytic site of plant SDHs was investigated based on our models of *V.vinifera* SDH homologs.

Nineteen putative active site residues (36C, 38S, 39D, 42H, 48C, 49A, 51F, 61H, 62E, 110F, 112G, 113S, 147E, 148P, 151V, 268L, 291F, 292R and 293Y; numbering in LIDH(Q1PSI9) without the first 20 amino acids) were identified either coordinating the zinc ion or forming potential non-covalent interactions with NAD(H) and L-idonate. Ten out of the 19 residues were considered strictly conserved throughout all plant SDH forms, and six additional residues are also largely conserved with variations in only a few SDH sequences (Figure 3). These observations revealed a potential structural basis for the preserved function of plant SDHs. Interestingly, three other residues were found to be uniformly exchanged (Y42H, A112G and T113S) between core Eudicot SDH Class I and Class II while monocot and *A. coerulea* SDHs resemble SDH Class I at these amino acid sites (Figure 3). A closer inspection of these residues showed that the oxygen atom of C5 hydroxyl of L-idonate was poised to potentially interact with both 42H and 113S within distances of 4 Å and 2.6 Å respectively (Figure 5C). Additionally, the oxygen atom of the C6 ketone group of L-idonate was within non-covalent interaction distance to 113S (3.5 Å; Figure 5C). Notably, the replacement of 42Y (hydrophobic aromatic side chain) with 42H (charged side chain) in LIDH has the potential to change the hydrophobicity in the substrate-binding pocket (Figure 5D), which may lead to the preferential binding of L-idonate over D-sorbitol. These observations potentially provided a structural explanation for the unique activity of *V. vinifera* LIDH compared to other plant SDHs. Previous studies have indicated that the chiral configuration at C5 is not a determining factor for SDH substrate specificity [18,20], however, our analysis suggested that the C5 hydroxyl group and the C6 ketone group of L-idonate potentially affect substrate binding affinity due to amino acid substitutions at 42H, 112G and 113S in Class II SDHs. A previously identified SDH from apple fruit [9] was found to be the single Class II SDH (MDP0000305455) in *M. domestica* in the present study. This SDH has a much lower affinity for sorbitol (K_m_ 247 mM [9]) compared to other SDHs purified (K_m_ 40.3 mM [76], 86.0 mM [7]) or cloned (K_m_ 83.0 mM [10]; SDH Class I) from apple species. While the kinetic differences were suggested to be due to protein configuration changes between the fusion protein and native protein [9], the present analysis indicated that they might have been be due also to amino acid substitutions at the catalytic site.

From an evolutionary point of view, amino acid changes leading to the shift of enzyme substrate specificity are usually derived from positive Darwinian selection after gene duplication [41,43]. Results from the natural selection analyses in the present study are consistent with this suggestion. The three amino acid sites (42H, 112G and 113S) displaying substitutions between SDH Class I and Class II are all under positive natural selection (Table 3). At the moment, the enzymatic characterization of plant SDH is still fragmentary; no information is available regarding plant SDH activity with L-idonate, except for the activity of *V. vinifera* LIDH [48]. Site mutation and enzymatic studies are currently underway in our laboratory to investigate this hypothesis.

### Meta-analysis of sorbitol dehydrogenase related gene expression

**Figure 6:**
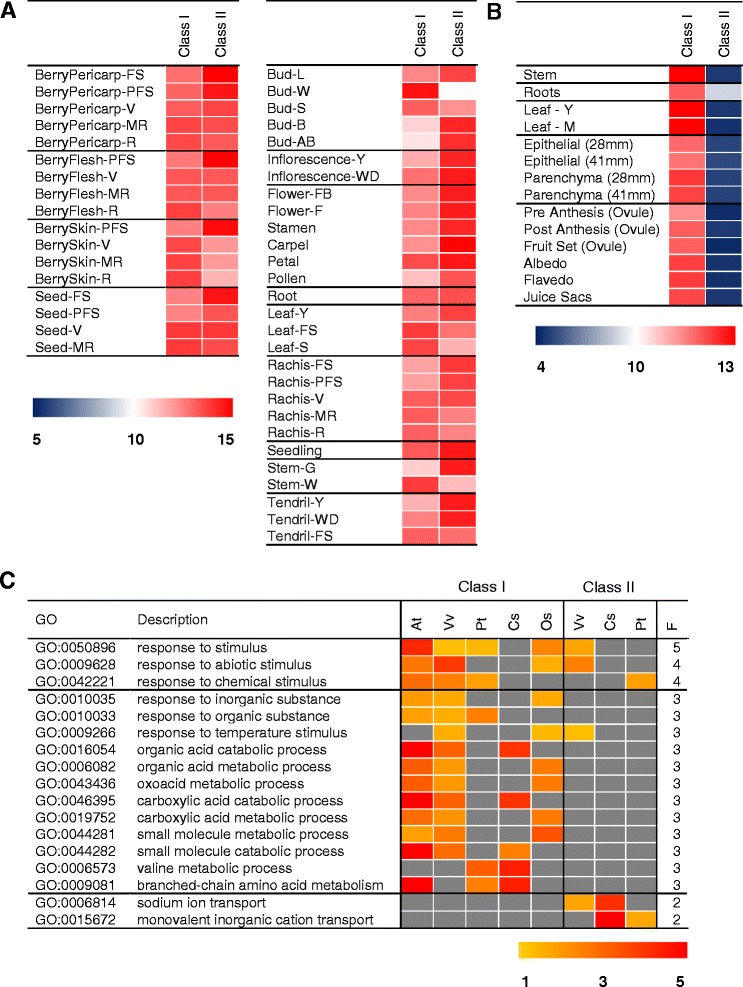
Transcript and gene co-expression profiles of SDH in different plants. **A**. Expression profiles for Class I and Class II SDH genes in various tissues and developmental stages of *V. vinifera*. Class I and II SDH genes were moderately to highly expressed in most tissues (Log2 intensity > 10; 50th percentile of all gene expression values, see Methods). The heatmap was adjusted to colour ranges between log2 intensity of 5 (blue), 10 (white) and 15 (red) to illustrate low, moderate and high expression when compared to all other genes respectively. **B**. Expression profiles for Class I and Class II SDH gene in citrus. The heatmap was adjusted to colour ranges between log2 intensity of 4 (blue), 10 (white) and 14 (red) to illustrate low, moderate and high expression when compared to all other genes respectively. **C**. Heatmap of selected enriched GO terms (−log10 (adj. *p*-value) for genes co-expressed with SDHs from *A. thaliana* (At), *V. vinifera* (Vv), *C. sinensis* (Cs), *P .trichocarpa* [84], *O. sativa* (Os) and associated frequencies in the plants tested. Light and dark orange denote enrichment scores between 1 and 3 respectively. Highly enriched scores (>5) are coloured in red. Grey colour denotes no significant enrichment.

In addition to changes in enzyme activity, gene evolution after duplication can also occur at the transcriptional level [42]. Expression division appears to be more common than structural evolution and often occurs rapidly after gene duplication [42,78,79]. To further characterize the evolutionary pattern of plant SDH genes and also to explore the role of SDH related genes during plant development, a survey of transcriptional data was undertaken. Based on the availability of microarray and RNA sequencing data and the presence of both classes of SDH in the genome, grapevine and citrus species were selected. In addition, the expression profile of the single Class I SDH (AT5G51970, Figure 2) in *A. thaliana* was used as a model reference [80]. This gene was highly expressed in cotyledons, leaves and late stages of seed development compared to organs such as flowers (stamen, petal, carpel) and shoots (inflorescence, vegetative, transition), where it was marginally expressed (data not shown).The results support a potential role for SDH Class I during seed germination in *A. thaliana* [23], soybean [37] and maize [8,38]. In grapevines, transcriptional patterns of VIT_16s0100g00290 (SDH Class II, LIDH) and VIT_16s0100g00300 (SDH Class I, SDH) were analysed using the normalised grapevine gene expression atlas of the ‘Corvina’ cultivar [81]. Notable differences in gene expression intensities and dynamics were observed between SDH Class I and Class II (Figure 6A; Additional file 11: Table S1). The transcript abundance of grapevine SDH Class I was highest in the ripening stages of berries (measured in pericarp, pulp, seeds and skins), resembling the expression profiles reported for Class I SDHs in apple [10,27,29]. In most cases, transcript abundance was lowest in young berry growth stages and increased gradually until harvest in berry tissues. Developmental up-regulation of SDH Class I transcripts in other cultivars such as ‘Shiraz’ [82] and ‘Tempranillo’ [83] during berry development under normal conditions was also evident. In addition, the latter work showed sorbitol is present in leaves and berries, and that the biochemical activity of SDH Class I, involving sorbitol oxidation, coincided with SDH class I transcripts levels in these berries during development [83]. Similarly, developmental increases of the grapevine SDH Class I transcript were observed in leaf, rachis, seed and tendrils. Interestingly, gene expression of grapevine SDH Class I was highly induced in winter buds and followed a gradual down-regulation during dormancy release. A similar gene expression and protein activity pattern reported in raspberry [84] and pear [39] respectively may reflect a response to the environment where dormancy periods encompasses dehydration and temperature (cold) stress, although developmental processes could take place concurrently. Taken together, this suggests an active role for SDH Class I in developmental processes through the coordinated regulation of transcript and protein activities in controlling the flux of sorbitol (and related polyols) in grapevines which may be critical in maintaining cell and tissue homeostasis in the mature tissues [83] where oxidative stress is inherent [85,86].

Expression profiles of SDH Class II were well represented in most grapevine organs with the highest expression in berries at fruit-set and in flower carpels. A striking developmental down-regulation of grapevine SDH Class II genes was evident in most grapevine organs, where expression levels in young tissues of berries (pericarp, flesh, skin and seed), buds, leaves, stems and tendrils were high and gradually decreased during development (Figure 6A). We have previously demonstrated in a cross-comparison study involving RNA-seq, microarray and qRT-PCR in young, early veraison, late veraison and ripening berries of grapevine [82] that SDH Class II genes were developmentally down-regulated consistently in all profiling platforms. This distinct expression coincides with the accumulation of TA biosynthesis in young/immature tissues [48,87].

In citrus, SDH Class I and SDH Class II genes were represented by probesets “Cit.9778.1.S1_s_at” and “Cit.9780.1.S1_s_at” respectively. Although gene expression studies encompassing developmental series in citrus are not as comprehensive compared to *A. thaliana* and grapevine, several striking observations could be inferred (Figure 6B; Additional file 11: Table S1). The citrus SDH Class I gene was highly expressed regardless of organ and tissue, including stems, roots, leaves, ovules and fruit tissues (albedo, flavedo, juice sacs), similar to that of grapevine SDH Class I. Interestingly, SDH Class II genes were expressed to a very low level (possibly in fact not at all) in the majority of organs, including fruit tissues, except for the root where expression was highest. It is speculated that this may reflect the trace amount of TA detected in fruits of sweet oranges and other citrus species [63]. Until now, no information, to our knowledge, has been reported on the function of citrus SDHs. Given the novel transcription profiles of one the two citrus Class II SDHs (specifically expressed in root tissues), and the presence of an additional Class II SDH (albeit this sequence was not represented in the array from which these data were analysed), these features may indicate a novel function of SDHs specific to root tissues of sweet oranges and therefore, deserve more attention in future research. In addition to *V. vinifera* and citrus, divergent transcription profiles have also been reported for SDHs from apple [10] and pear [39] where the single copy Class II SDH genes were shown to be under independent transcriptional regulation from other SDH genes. Taken together, divergent expression profiles for SDH Class I and SDH Class II appear to be true to all species where two classes are present, supporting a gene functional divergence at the expression level.

### Gene co-expression mining in various plant species

Gene co-expression network analysis (GCA) is based on the principle that genes involved in similar and/or related biological processes may be expressed in a proportional manner, thereby providing a unique tool to understand gene function. Based on information availability, co-expressed gene lists of SDHs from *A. thaliana*, rice, poplar, grapevine and citrus (Additional file 11: Table S2-S9) were retrieved from publicly available co-expression databases [88-90]. In *A. thaliana*, the SDH Class I homologue (At5g51970) was significantly co-expressed with 67 genes (33% of total genes in the list) involved in branched chain amino acid metabolism, 72 genes (36%) involved in response to various stimuli, 37 genes (19%) involved in protein import in the peroxisome and 17 genes (9%) involved in auxin metabolism (Additional file 11: Table S2). In grapevines, the SDH Class I homologue (VIT_16s0100g00300) was significantly co-expressed with genes involved in abiotic stress (21%), peptide metabolism (13%) and lipid metabolism (13%) (Additional file 11: Table S3; Additional file 12: Table S2–S3). The co-expression results presented here corroborated with recent findings that the importance of SDH Class I lies in regulating sorbitol levels via its biochemical activity and gene expression during various abiotic stresses [83]. More importantly, intracellular accumulation of sorbitol to high levels, accentuated under salt and osmotic stress, significantly reduced stress-induced biomass loss of grapevine berry cell suspensions which were likely the results of the polyol utilisation as an effective osmoprotectant and cellular homeostasis buffer [83]. Similar to its Arabidopsis counterpart (At5g51970), it is therefore likely that grapevine SDH Class I plays an important role in abiotic stress tolerance via the synergistic regulation of polyol transport and metabolism. The SDH Class II homologue (LIDH, VIT_16s0100g00290) was also significantly co-expressed with genes related to abiotic stress response (35%). Other genes related to hexose biosynthetic pathways and carbohydrate metabolism (25%), protein biogenesis and catabolism (8%) and malic acid transport (6%) were also evident in the list of co-expressed genes (Additional file 11: Table S4). GO terms associated with these genes were also enriched within the gene lists (FDR < 0.05). Interestingly, GO enrichment analysis of co-expressed genes showed that terms associated with “malate trans-membrane transport” and “response to abiotic stimulus” were highly enriched (FDR < 1.51E-04 and 3.5E–03 respectively) (Additional file 12: Table S2). Similarly to the grapevine SDH Class I gene, SDH Class II transcription was also stress responsive, being down-regulated during the heat stress recovery of grapevine leaves and up-regulated during exposure to UV-C light irradiation (Additional file 12: Table S3). Based on our coexpression analysis, we speculate that the involvement of Class II SDHs in abiotic stress responses is likely to occur via a separate mechanism from that of sorbitol metabolism, namely the ascorbate-glutathione cycle [91] and specifically in regulating the balance between the biosynthesis of ascorbate by the L-galactose pathway [92] and its catabolism. This is supported in part in grapevines in which a marked down-regulation of SDH Class II (LIDH) protein (impeding TA formation) and the up-regulation of proteins involved in L-galactose pathway (favouring Asc formation) in shoots of grapevines during drought stress were observed [93]. Therefore, the stress responsive nature of SDH Class II gene and enzyme could potentially function as an extra level of control (preventing loss of Asc to TA). The *C. sinensis* SDH Class II gene (Cit.9780.1.S1_at) was significantly co-expressed with genes involved in ion transport (11%), ubiquinone biosynthesis/oxidative phosphorylation (20%) and ribosome biogenesis (9%) (Additional file 11: Table S6). GO terms associated with these genes were highly enriched within the co-expressed gene lists (Additional file 12: Table S5). Unlike Class I SDHs, enriched GO terms associated with Class II SDH co-expressed genes were more specialised to each corresponding plant but shared a common set of co-expressed genes related to transporters (Additional file 11: Table S7; Additional file 12: Table S6). In rice, the top 200 genes co-expressed with SDH (Os08g0545200) were primarily enriched for genes involved in stress response (31%), carboxylic acid biosynthesis (16%), plastid organisation (11%), protein transport (10%) and starch metabolism (5%) (Additional file 11: Table S5; Additional file 12: Table S4).

Enriched GO parent terms such as “response to stimulus” and descendent terms “response to abiotic stimulus”, were frequently enriched in SDH Class I co-expressed lists and slightly in SDH Class II containing plant species (Figure 6C; Additional file 12: Table S1-S9). These observations agreed with previous reports that SDHs (Class I) in *A. thaliana* [13,23] and grapevine [83] play an active role during drought stress and recovery processes and also suggest some shared functions related to stress tolerance between the two classes of SDH, even though to a conservative degree and potentially involving a separate mechanistic route. Therefore, enriched GO parent terms associated with “organic acid metabolic process” and “branched-chain amino acid metabolism” were demonstrated to be more relevant to SDH Class I co-expressed genes but not to SDH Class II (Figure 6C). This is not surprising as response to various stresses involves the coordinated regulation of amino acid and polyol accumulation [94]. On the other hand, co-expression analysis showed that plant SDH Class II could be tightly linked to mechanisms related to transport and compartmentation of cations and solutes (Figure 6C). In membrane transport and compartmentation systems involving pumps, carriers and ion channels are also pivotal for ion homeostasis and equivocally involved in a wide range of stress conditions [95]. In addition, divergent co-expression profiles across species have also been observed for both classes of SDH. In general, monocot rice SDH-related genes have more common co-expression responses with core Eudicot SDH Class I than with SDH Class II, corresponding with the finding that monocot SDH has a closer relationship with core Eudicot SDH Class I than SDH Class II at the enzyme structural level.

## Conclusions

SDH is the key enzyme involved in sorbitol metabolism in higher plants. The results of the present study demonstrated that core Eudicot SDHs have evolved into two distinct lineages: SDH Class I and SDH Class II. Class I SDH genes were present in all core Eudicot species investigated in this study and appear to be essential for the normal growth of plants. Class II SDH genes were found to be absent in *Brassicaceae*, *Leguminosae*, most *Asterid*s (except *S. tuberosum*) and some other plants. The previously characterized LIDH involved in TA synthesis in *V. vinifera* has now been identified as a Class II SDH and represents a novel function of SDH genes in *V. vinifera*. The role of LIDH in TA synthesis may be relevant to the function of Class II SDHs in other species. Phylogeny, natural selection and genomic structure analyses supported the emergence of SDH Class II as a result of positive natural selection after tandem duplication, which might occur in the common ancestor of core Eudicot plants. Furthermore, positive natural selection has only acted on specific amino acid sites in the SDH Class II lineage. Protein modelling analyses revealed substitutions of three putative active site residues for Class I and Class II SDHs, which may be responsible for the unique enzyme activity of *V. vinifera* LIDH. Gene expression analysis demonstrated a clear transcriptional divergence between SDH Class I and Class II in several plants and supports the divergence of Class II SDHs at the expression level as well. Future work should be dedicated to uncovering the enzymatic activities and roles of Class II SDH gene products in plant metabolism.

## Methods

### Identification of sorbitol dehydrogenase homologous genes in higher plants

To identify homologous SDHs in angiosperm plants, the amino acid sequence of *A. thaliana* SDH (accession no. At5g51970) was used as a query to BLAST against the genomes of angiosperm species at Phytozome (http://www.phytozome.net/), with the exception of *M. domestica* for which genome dataset at Plant Genome Duplication Database (PGDD, http://chibba.agtec.uga.edu/duplication/) was used instead. To increase dataset coverage, the genomes of 8 recently sequenced species including *Cajanus cajan*, *Jatropha curcas*, *Capsicum annuum*, *Brassica oleracea*, *Eutrema saisugineum*, *P. mume*, *Hordeum vulgare* and *Aegilops tauschii* were also queried using the corresponding genome databases. BLAST hits with an expectancy value (E value) of zero were selected as SDH homologs were subjected to another round of BLAST searches within the genomes from which they were identified. Only the primary transcript was chosen when alternative transcripts occurred. In addition, five partial SDH protein sequences of *P. bretschneideri* [39] and one SDH sequence of *Eriobotrya japonica* [35] were obtained from literature searches. Homologous SDHs of *P. hortorum* were provided by the *P. hortorum* genome sequencing project author (Prof. Robert K. Jansen, The University of Texas at Austin).

### Phylogenetic analysis of sorbitol dehydrogenase

The Uniprot database was queried for previously identified MDR mammal SDHs and yeast SDHs. Only reviewed entries were selected and used as the out-group in this phylogenetic analysis. Multiple sequence alignments of 102 sequences (92 plant SDHs, 7 mammal SDHs and 3 yeast SDHs) were carried out using ClustalW2 [96]. The evolutionary distances of target SDHs (pairwise p-distance) were estimated using MEGA6 software [97]. The Neighbour Joining tree was inferred by MEGA6 software [97] using the p-distance [98] substitution model, the certainty at each node was assessed by the Interior-branch Test method (1000 times iteration). Maximum likelihood trees were estimated by MEGA6 software [97] using the JTT+GAMMA substitution model [99], the best fitting model as determined by the “Find Best DNA/Protein Models” function in MEGA6. Bootstrap supports for Maximum likelihood trees were calculated from 1000 replicates. For both Neighbour Joining and Maximum likelihood methods, the Gaps/Missing Data Treatment parameter was set as Complete-Deletion to eliminate the effects of gaps and insertions. The developed phylogenetic trees were rooted on the yeast SDHs and annotated using the FigTree version 1.4.2 software (http://tree.bio.ed.ac.uk/software/figtree/).

### Sequence alignment and protein subdomain analysis

Preliminary sequence identity of SDHs was obtained by local all-vs-all BLAST using NCBI-BLAST-2.2.29 tool [100] downloaded from ftp://ftp.ncbi.nlm.nih.gov/blast/executables/blast+/LATEST/. The BLAST results were sorted according to respective phylogeny groups. Average pair-wise sequence identities were calculated using Microsoft Excel software based on the BLAST results. Protein functional domains were predicted using InterPro (http://www.ebi.ac.uk/interpro/). Secondary structure analysis was implemented with ESPript3.0 tool (http://espript.ibcp.fr/ESPript/ESPript/) using human SDH (PDB: 1PL8) as a template. All residue numberings in the present study are according to LIDH (Q1PSI9) without the first 20 amino acids (unless otherwise declared) which was predicted to be a mitochondria-targeting signal sequence (data not shown; alignment corresponding to this region was highly divergent).

### Gene duplication pattern characterization and synteny analysis

The MCScanX package [101] from http://chibba.pgml.uga.edu/mcscan2/ was employed to investigate gene duplication patterns of plant SDHs. In order to elaborate on the origin of the core Eudicot Class II SDHs, plant genomes containing SDHs from both Class I and Class II were selected. These were further refined to genomes for which predicted genes have been mapped into corresponding chromosome locations. *A.thaliana* was included as a reference for inter-species collinear block analysis. Amino acid sequence files and gene position files were downloaded either from PGDD or from Phytozome databases and were further modified to suit the requirements of the MCScanX software. BLAST tool NCBI-BLAST-2.2.29 [100] was used for intra and inter species genome comparisons. The E-value threshold was set at 10^-5^ for all analyses. For gene duplication pattern identification, self-genome all-vs-all BLAST was performed. The *duplicate_gene_class ifier* program from the MCScanX package was applied to each dataset. For collinear SDH gene pair identification, amino acid sequences and genetic position information of chromosomes containing SDHs were extracted from each species, then combined to perform the multi-species MCScanX analysis. The SDH gene family file was created manually by including all the SDHs identified from the selected species. The *family_circle_plotter.java* tool at MCScanX package was used to display the results.

### Natural selection analysis

Natural selective pressure on plant SDH was examined by measuring the ratio of non-synonymous to synonymous substitutions (dN/dS=w). Codon-based maximum-likelihood estimates of w was performed using codeml in PAML4.7 [73]. Multiple-alignment of conserved domain sequences (CDS) for those identified plant SDHs was carried out using ClustalW2 [96]. Significant insertions and gaps were removed manually. To facilitate the input data requirements of codeml, an additional Maximum Likelihood tree was constructed using a smaller dataset where SDHs with no CDS sequence available were removed. The sub-tree covering the plant SDHs was used in codeml. Branch pattern specification was implemented using Treeview1.6.6 (http://taxonomy.zoology.gla.ac.uk/rod/treeview.html). Four target clades were specified based on the present phylogenetic analysis: monocot SDH, *A. coerulea* SDH, core Eudicot SDH Class I and core Eudicot SDH Class II. The w values for these clades were represented as w[mono], w[Aer], w[sdhC1] and w[sdhC2] respectively. Nested likelihood ratio tests(LRTs) were performed to assess the significance of the model under different hypothesises: (w[mono]≠w[Aer]≠w[sdhC1]=w[sdhC2], w[mono]=w[Aer]≠w[sdhC1]≠w[sdhC2], w[mono]≠w[Aer]≠w[sdhC1]≠w[sdhC2], w[mono]=w[Aer]=w[sdhC1]≠w[sdhC2], w[mono]=w[Aer]=w[sdhC1]≠w[sdhC2] with w[sdhC2]=1). The corresponding p values were calculated using the online tool at http://graphpad.com/quickcalcs/PValue1.cfm. In the Site-specific model M1, two site classes were specified: highly conserved sites (w0) and neutral sites (w1=1). For the Site-specific model M2, there were three site classes: highly conserved sites (w0), neutral sites (w1=1) and positively selected sites (w2). For w assessments with the Branch-site models, core Eudicot SDH Class II was specified as the foreground group. In the Branch-site model A, four site classes were specified. The first two classes have w ratios of w0 and w1 respectively, corresponding to highly conserved sites and neutral sites across all lineages. In the other two site classes, the background lineages have w0 or w1 while the foreground lineages have w2.

### Ancestral sequence reconstruction and evolution rate analyses

The ancestral sequence (amino acid) reconstruction for the internal nodes of the obtained plant SDH phylogeny was carried out using codeml in PAML4.7 [73]. The Empirical_Frequency model, which allowed the estimates of the stationary frequencies based on user dataset, was performed on the plant SDHs. Ancestral amino acid sequences for nodes representing monocot SDH, *A. coerulea* SDH, core Eudicot SDH Class I and core Eudicot SDH Class II were used for Tajima’s RRT analysis [74] using MEGA6.0 software [97].

### Protein structure modelling analysis

SDH homology modelling was carried out using ICM Pro (Molsoft LLC, La Jolla, CA, USA). Models of *V. vinifera* LIDH (Uniprot ID: Q1PSI9; accession no: GSVIVT01010646001) and *V. vinifera* SDH (Uniprot ID: D7TMY3; accession no: GSVIVT01010642001) structures were generated with the human SDH (PDB:1PL8) as a template. Given that no plant SDH structures exist in the protein data bank we chose the model with the highest identity as performed within the Molsoft software package. Ligands including the zinc atom, NAD^+^, D-sorbitol and L-idonate were docked into the models using the Molsoft Monte Carlo method [102]. Residues within 5 Å to the ligands were inspected for enzyme-ligand interaction potential. All molecular visualizations were obtained using the PyMOL graphic tool (The PyMOL molecular graphics system, Version 1.3r1. Schrodinger, LLC). The deduced reaction mechanism of *V. vinifera* LIDH on the oxidation of L-idonate was created using the Marvin online tool (http://www.chemaxon.com/marvin/sketch/index.php). Protein hydrophobicity profiles were implemented in PyMOL using the Color_h script (http://www.pymolwiki.org/index.php/Color_h), based on the hydrophobicity scale defined at http://us.expasy.org/tools/pscale/Hphob.Eisenberg.html. All residue numberings are according to LIDH (Q1PSI9) without the first 20 amino acids.

### Meta-analysis of developmental gene expression

Identification of corresponding probesets in the microarray platforms of *A. thaliana*, rice, poplar, grapevine and citrus were performed using the BLAST software (NCBI-BLAST-2.2.29+) [100], and grapevine Class I (VIT_16s0100g00290) and Class II (VIT_16s0100g00290) SDH sequences with default settings. The top hits for each corresponding probeset in the microarray platform of each species were selected for downstream analysis (Additional file 11). Normalised gene expression atlases encompassing transcriptional data during growth and development of *A. thaliana*, grapevine and citrus were retrieved from the Botany Array Resource (BAR) [80], *Vitis* co-expression database (VTCdb) [88] and Network inference of citrus co-expression (NiCCE) [89] webservers, respectively. Only experimental conditions relating to tissue/organ development and probesets intensities (normalised) corresponding to Class I and Class II SDHs were retained. Normalised log2 intensities were deemed highly, well and lowly/not expressed when the intensities of total background distribution > 95th, at the 50th and < 20th percentile respectively.

### Gene co-expression mining in various plant species

Information on co-expressed genes with Class I and Class II SDHs in plants such as *A. thaliana*, poplar and rice (version 7.1) [90], grapevine (version 2.1) [88] and citrus [89] were retrieved from the various plant gene co-expression webservers. The top 200 co-expressed genes (unless otherwise specified) for each SDH class in each species were empirically chosen as a cut-off for significant co-expression, and to provide comparisons of enriched gene ontology (GO) terms within the co-expressed gene lists from each species. Enrichment of GO terms (i.e. biological processes, BP; molecular function, MF; cellular component, CC) were evaluated by hypergeometric distribution, adjusted by false discovery rate (FDR) for multiple hypothesis correction and using the ‘gProfileR’ package [103] in R (http://www.r-project.org) which interfaces g:profiler webserver (http://gprofiler.at.mt.ut.ee/gprofiler/). The ‘ordered query’ option was enabled to perform incremental enrichment analysis, which prioritises highly co-expressed genes and results in better functional GO term associations. GO terms were considered to be significantly enriched when FDR < 0.05 and > 2 genes were annotated with the same GO term. Enriched GO terms from the SDH co-expressed gene lists across tested plants (*A. thaliana*, poplar, rice, grapevine and citrus), were considered ‘commonly occurring’ when more than 3 counts were present for each enriched GO term.

## Availability of supporting data

All relevant supporting data can be found within the additional files accompanying this article. Phylogenetic data supporting the results of this article are available in the TreeBASE repository at http://purl.org/phylo/treebase/phylows/study/TB2:S17300.

## Additional files

Additional file 1:
**Displays the molecular structures of SDH substrates.**
Additional file 2: Table S1. Contains SDH gene IDs from corresponding species and organisms. **Table S2.** Contains pairwise p-distance values of SDH sequences. **Table S3.** Contains information on sequence renaming. **Table S4.** Contains the all-vs-all BLAST results of SDH amino acid sequences. Table S5 contain the identified collinear SDH gene pairs. Additional file 3:
**Contains the original amino acid sequences of the identified plant, mammal and yeast SDHs.**
Additional file 4:
**Displays the complete Neighbour Joining tree for Figure** 2 **A.**
Additional file 5:
**Displays complete sequence alignment for Figure** 3. Additional file 6:
**Contains gene duplication pattern information.** Tables “cs”, “eg”, “md”, “pm”, “pp”, “pt”, “st”, “tc”, “vv” refer to *C. sinensis*, *E. grandis*, *M. domestica*, *P. mume*, *P. persica*, *P. trichocarpa*, *S. tuberosum*, *T. cacao* and *V. vinifera* respectively. Additional file 7:
**Contains input and output data for natural selection modelling analyses.** “-output” files are codeml outputs and are recommended to be viewed using Microsoft WordPad. “.phy” is phylogenetic tree file and can be viewed using Treeview software. “.ctl” is a control file and can be viewed using any text viewer. “sdh-pep2.fas” sequence file was produced from Additional file 3 by manually removing the significant gaps, insertions; sequences with no CDS sequence available were also removed. “sdh-cds2.fas” is the corresponding CDS sequences for “sdh-pep2.fas”. “sdh-pep2.nwk” is the phylogenetic tree produced from “sdh-cds2.fas” and can be viewed using any phylogenetic tree viewer software. Sequence IDs are represented by numbers for software input convenience (see Additional file 2: Table S3 for sequence ID renaming information). Amino acid site numbering is according to LIDH (Uniprot No: Q1PSI9) without the first 20 amino acids. Additional file 8:
**Contains input and output data for the reconstruction of ancestral SDH sequences.** “sdh-pep.fas” contains amino acid sequences for the plant SDH sub-branch. The “ancestral-sequence-construction_output” file is codeml output and can be viewed using any text viewer. Ancestral sequences for corresponding branches were extracted and put in the “interpreted-ancestral-sequence.fas” file for readers’ convenience. Additional file 9:
**Contains the Tajima’s RRT test outputs.**
Additional file 10:
**Contains the modelled structures files of Vv_LIDH and Vv_SDH and additional illustration figures.** “Asp195_NAD.png” displays the interaction of Asp195 with the hydroxyl groups at C1 and C2 of L-idonate. “LIDH-hydrophobicity.png” and “SDH-hydrophobicity.png” display the overall hydrophobicity profiles of Vv_LIDH and Vv_SDH respectively. Amino acid site numbering is according to LIDH (Uniprot No: Q1PSI9) without the first 20 amino acids. Additional file 11:
**Contains a Microsoft Excel spread sheet with detailed results of transcript and gene co-expression analysis of Class I and Class II SDH in plants.** Table S1 contains gene expression profile of Class I and Class II SDH profile in various tissues of (A) grapevine and (B) sweet oranges. Table S2 – S9 contains lists of all significantly co-expressed genes and respective rank, function description, and co-expression metric with class I and II SDH in *A. thaliana* (Table S2), grapevine (Table S3 and S4), rice (Table S5), sweet orange (Table S6 and S7) and poplar (Table S8 and S9). Additional file 12:
**Contains a Microsoft Excel spread sheet with detailed results of functional (GO) enrichment analysis of significantly co-expressesed genes of class I and II SDH in plants.** Table S1 – S8 contains outputs of GO enrichment analysis containing enriched GO ID, description, adjusted p-value, and lists of genes having the enriched GO term for A. thaliana (Table S1), grapevine (Table S2 and S3), rice (Table S4), sweet orange (Table S5 and S6) and poplar (Table S7 and S8). Table S9 contains a summary of common enriched GO ID/term identified among the co-expressed genes with SDHs in the aforementioned plants tested.

## Abbreviations

SDH: Sorbitol dehydrogenase
LIDH: L-idonate-5-dehydrogenase
TA: Tartaric acid
MDR: Medium-chain dehydrogenase/reductase
ADH: Alcohol dehydrogenase
5KGA: 5-keto-D-gluconate
Mya: Million years ago
WGD: Whole genome duplication
RRT: Relative rate tests
GCA: Gene co-expression network analysis
PGDD: Plant Genome Duplication Database
CDS: Conserved domain sequences
LRTs: Likelihood ratio tests
BAR: Botany Array Resource
VTCdb: *Vitis* co-expression database
NiCCE: Network inference of citrus co-expression
GO: Gene ontology
BP: Biological processes
Asc: Ascorbate
MF: Molecular function
CC: Cellular component
FDR: False discovery rate

## References

[1] Iwata T, Hoog JO, Reddy VN, Carper D (1993). Cloning of the human Sorbitol Dehydrogenase gene. Invest Ophth Vis Sci.

[2] Karlsson C, Jornvall H, Hoog JO (1991). Sorbitol Dehydrogenase - cDNA coding for the rat enzyme - variations within the Alcohol-Dehydrogenase family independent of quaternary structure and metal content. Eur J Biochem.

[3] Wang T, Hou M, Zhao N, Chen Y, Lv Y, Li Z (2013). Cloning and expression of the sorbitol dehydrogenase gene during embryonic development and temperature stress in *Artemia sinica*. Gene.

[4] Niimi T, Yamashita O, Yaginuma T (1993). A cold-inducible Bombyx gene encoding a protein similar to mammalian Sorbitol Dehydrogenase - yolk nuclei-dependent gene-expression in diapause eggs. Eur J Biochem.

[5] Sarthy AV, Schopp C, Idler KB (1994). Cloning and sequence determination of the gene encoding Sorbitol Dehydrogenase from *Saccharomyces cerevisiae*. Gene.

[6] Ng K, Ye RQ, Wu XC, Wong SL (1992). Sorbitol Dehydrogenase from *Bacillus subtilis* - purification, characterization, and gene cloning. J Biol Chem.

[7] Negm FB, Loescher WH (1979). Detection and characterization of Sorbitol Dehydrogenase from apple callus-tissue. Plant Physiol.

[8] Doehlert DC (1987). Ketose reductase-activity in developing maize endosperm. Plant Physiol.

[9] Yamada K, Oura Y, Mori H, Yamaki S (1998). Cloning of NAD-dependent sorbitol dehydrogenase from apple fruit and gene expression. Plant Cell Physiol.

[10] Park SW, Song KJ, Kim MY, Hwang JH, Shin YU, Kim WC (2002). Molecular cloning and characterization of four cDNAs encoding the isoforms of NAD-dependent sorbitol dehydrogenase from the Fuji apple. Plant Sci.

[11] Ohta K, Moriguchi R, Kanahama K, Yamaki S, Kanayama Y (2005). Molecular evidence of sorbitol dehydrogenase in tomato, a non-Rosaceae plant. Phytochemistry.

[12] Sutsawat D, Yamada K, Shiratake K, Kanayama Y, Yamaki S (2008). Properties of sorbitol dehydrogenase in strawberry fruit and enhancement of the activity by fructose and auxin. J Jpn Soc Hortic Sci.

[13] Aquayo MF, Ampuero D, Mandujano P, Parada R, Muñoz R, Gallart M (2013). Sorbitol dehydrogenase is a cytosolic protein required for sorbitol metabolism in *Arabidopsis thaliana*. Plant Sci.

[14] Persson B, Zigler JS, Jornvall H (1994). A super-family of medium-chain dehydrogenases/reductases (MDR) - Sub-lines including zeta-crystallin, alcohol and polyol dehydrogenases, quinone oxidoreductases, enoyl reductases, Vat-1 and other proteins. Eur J Biochem.

[15] Persson B, Hedlund J, Jornvall H (2008). The MDR superfamily. Cell Mol Life Sci.

[16] Jornvall H, Persson M, Jeffery J (1981). Alcohol and polyol dehydrogenases are both divided into 2 protein types, and structural-properties cross-relate the different enzyme-activities within each type. P Natl Acad Sci-Biol.

[17] Nordling E, Jornvall H, Persson B (2002). Medium-chain dehydrogenases/reductases (MDR) - family characterizations including genome comparisons and active site modelling. Eur J Biochem.

[18] Lindstad RI, Koll P, McKinley-McKee JS (1998). Substrate specificity of sheep liver sorbitol dehydrogenase. J Biochemical.

[19] Lindstad RI, Hermansen LF, McKinley-Mckee JS (1992). The kinetic mechanism of sheep liver sorbitol dehydrogenase. Eur J Biochem.

[20] Oura Y, Yamada K, Shiratake K, Yamaki S (2000). Purification and characterization of a NAD(+)-dependent sorbitol dehydrogenase from Japanese pear fruit. Phytochemistry.

[21] Guo ZX, Pan TF, Li KT, Zhong FL, Lin L, Pan DM (2012). Cloning of NAD-SDH cDNA from plum fruit and its expression and characterization. Plant Physiol Biochem.

[22] Pauly TA, Ekstrom JL, Beebe DA, Chrunyk B, Cunningham D, Griffor M (2003). X-ray crystallographic and kinetic studies of human sorbitol dehydrogenase. Structure.

[23] Nosarzewski M, Downie AB, Wu B, Archbold DD (2012). The role of sorbitol dehydrogenase in *Arabidopsis thaliana*. Funct Plant Biol.

[24] Yancey PH, Clark ME, Hand SC, Bowlus RD, Somero GN (1982). Living with water-stress - evolution of osmolyte systems. Science.

[25] Loescher WH (1987). Physiology and metabolism of sugar alcohols in higher-plants. Physiol Plantarum.

[26] Loescher WH, Marlow GC, Kennedy RA (1982). Sorbitol metabolism and sink-source interconversions in developing apple leaves. Plant Physiol.

[27] Nosarszewski M, Clements AM, Downie AB, Archbold DD (2004). Sorbitol dehydrogenase expression and activity during apple fruit set and early development. Physiol Plantarum.

[28] Nosarzewski M, Archbold DD (2007). Tissue-specific expression of sorbitol dehydrogenase in apple fruit during early development. J Exp Bot.

[29] Wang XL, Xu YH, Peng CC, Fan RC, Gao XQ (2009). Ubiquitous distribution and different subcellular localization of sorbitol dehydrogenase in fruit and leaf of apple. J Exp Bot.

[30] Yamaguchi H, Kanayama Y, Soejima J, Yamaki S (1996). Changes in the amounts of the NAD-dependent sorbitol dehydrogenase and its involvement in the development of apple fruit. J Am Soc Hortic Sci.

[31] Wu BH, Li SH, Nosarzewski M, Archbold DD (2010). Sorbitol dehydrogenase gene expression and enzyme activity in apple: tissue specificity during bud development and response to rootstock vigor and growth manipulation. J Am Soc Hortic Sci.

[32] Iida M, Bantog NA, Yamada K, Shiratake K, Yamaki S (2004). Sorbitol- and other sugar-induced expressions of the NAD+−dependent sorbitol dehydrogenase gene in Japanese pear fruit. J Am Soc Hortic Sci.

[33] Kim HY, Ahn JC, Choi JH, Hwang B, Choi DW (2007). Expression and cloning of the full-length cDNA for sorbitol-6-phosphate dehydrogenase and NAD-dependent sorbitol dehydrogenase from pear (*Pyrus pyrifolia N.*). Sci Hortic.

[34] Bantog NA, Shiratake K, Yamaki S (1999). Changes in sugar content and sorbitol- and sucrose-related enzyme activities during development of loquat (*Eriobotrya japonica Lindl. cv. Mogi*) fruit. J Jpn Soc Hortic Sci.

[35] Bantog NA, Yamada K, Niwa N, Shiratake K, Yamaki S (2000). Gene expression of NAD(+)-dependent sorbitol dehydrogenase and NADP(+)-dependent sorbitol-6-phosphate dehydrogenase during development of loquat (*Eriobotrya japonica Lindl.*) fruit. J Jpn Soc Hortic Sci.

[36] Beruter J (1985). Sugar accumulation and changes in the activities of related enzymes during development of the apple fruit. J Plant Physiol.

[37] Kuo TM, Doehlert DC, Crawford CG (1990). Sugar metabolism in germinating soybean seeds - evidence for the sorbitol pathway in soybean axes. Plant Physiol.

[38] de Sousa SM, Paniago MD, Arruda P, Yunes JA (2008). Sugar levels modulate sorbitol dehydrogenase expression in maize. Plant Mol Biol.

[39] Ito A, Hayama H, Kashimura Y (2005). Partial cloning and expression analysis of genes encoding NAD(+)-dependent sorbitol dehydrogenase in pear bud during flower bud formation. Sci Hortic.

[40] Hartman MD, Figueroa CM, Piattoni CV, Iglesias AA (2014). Glucitol Dehydrogenase from peach (*Prunus persica*) fruits is regulated by thioredoxin h. Plant Cell Physiol.

[41] Flagel LE, Wendel JF (2009). Gene duplication and evolutionary novelty in plants. New Phytol.

[42] Zhang JZ (2003). Evolution by gene duplication: an update. Trends Ecol Evol.

[43] Hughes AL (1994). The evolution of functionally novel proteins after gene duplication. P Roy Soc B-Biol Sci.

[44] Hughes AL (2002). Adaptive evolution after gene duplication. Trends Genet.

[45] Hurles M (2004). Gene duplication: the genomic trade in spare parts. Plos Biol.

[46] Force A, Lynch M, Pickett FB, Amores A, Yan YL, Postlethwait J (1999). Preservation of duplicate genes by complementary, degenerative mutations. Genetics.

[47] Conant GC, Wolfe KH (2008). Turning a hobby into a job: how duplicated genes find new functions. Nat Rev Genet.

[48] DeBolt S, Cook DR, Ford CM (2006). L-Tartaric acid synthesis from vitamin C in higher plants. P Natl Acad Sci USA.

[49] Strommer J (2011). The plant ADH gene family. Plant J.

[50] Velasco R, Zharkikh A, Affourtit J, Dhingra A, Cestaro A, Kalyanaraman A (2010). The genome of the domesticated apple (*Malus x domestica Borkh.*). Nat Genet.

[51] Forney CF, Breen PJ (1985). Growth of strawberry fruit and sugar uptake of fruit disks at different inflorescence positions. Sci Hortic.

[52] Veitia RA, Bottani S, Birchler JA (2008). Cellular reactions to gene dosage imbalance: genomic, transcriptomic and proteomic effects. Trends Genet.

[53] Galdon BR, Mesa DR, Rodriguez EMR, Romero CD (2010). Influence of the cultivar on the organic acid and sugar composition of potatoes. J Sci Food Agric.

[54] Stafford HA (1959). Distribution of tartaric acid in the leaves of certain angiosperms. Am J Bot.

[55] Kramer EM (2009). *Aquilegia*: a new model for plant development, ecology, and evolution. Annu Rev Plant Biol.

[56] Worberg A, Quandt D, Barniske AM, Lohne C, Hilu KW, Borsch T (2007). Phylogeny of basal eudicots: insights from non-coding and rapidly evolving DNA. Org Divers Evol.

[57] Hoot SB, Magallon S, Crane PR (1999). Phylogeny of basal eudicots based on three molecular data sets: atpB, rbcL, and 18S nuclear ribosomal DNA sequences. Ann Mo Bot Gard.

[58] Moore MJ, Bell CD, Soltis PS, Soltis DE (2007). Using plastid genome-scale data to resolve enigmatic relationships among basal angiosperms. P Natl Acad Sci USA.

[59] Wang HC, Moore MJ, Soltis PS, Bell CD, Brockington SF, Alexandre R (2009). Rosid radiation and the rapid rise of angiosperm-dominated forests. P Natl Acad Sci USA.

[60] Bremer B, Bremer K, Chase MW, Fay MF, Reveal JL, Soltis DE (2009). An update of the angiosperm phylogeny group classification for the orders and families of flowering plants: APG III. Bot J Linn Soc.

[61] Roulin A, Auer PL, Libault M, Schlueter J, Farmer A, May G (2013). The fate of duplicated genes in a polyploid plant genome. Plant J.

[62] Saito K, Loewus FA (1989). Formation of tartaric acid in Vitaceous plants - relative contributions of L-ascorbic acid-inclusive and acid-noninclusive pathways. Plant Cell Physiol.

[63] Nour V, Trandafir I, Ionica ME (2010). HPLC organic acid analysis in different citrus juices under reversed phase conditions. Not Bot Horti Agrobo.

[64] Hudina M, Stampar F (2000). Sugars and organic acids contents of European (*Pyrus communis L.*) and Asian (*Pyrus serotina Rehd.*) pear cultivars. Acta Aliment Hung.

[65] Sha SF, Li JC, Wu J, Zhang SL (2011). Characteristics of organic acids in the fruit of different pear species. Afr J Agr Res.

[66] Fuleki T, Pelayo E, Palabay RB (1995). Carboxylic-acid composition of varietal juices produced from fresh and stored apples. J Agr Food Chem.

[67] Suarez MH, Rodriguez ER, Romero CD (2008). Analysis of organic acid content in cultivars of tomato harvested in Tenerife. Eur Food Res Technol.

[68] Ina Y (1996). Pattern of synonymous and nonsynonymous substitutions: an indicator of mechanisms of molecular evolution. J Genet.

[69] Kimura M (1977). Preponderance of synonymous changes as evidence for the neutral theory of molecular evolution. Letters to Nature.

[70] Yang ZH (1997). PAML: a program package for phylogenetic analysis by maximum likelihood. Comput Appl Biosci.

[71] Worth CL, Gong S, Blundell TL (2009). Structural and functional constraints in the evolution of protein families. Nat Rev Mol Cell Bio.

[72] Yang ZH, Nielsen R (2002). Codon-substitution models for detecting molecular adaptation at individual sites along specific lineages. Mol Biol Evol.

[73] Yang ZH (2007). PAML 4: phylogenetic analysis by maximum likelihood. Mol Biol Evol.

[74] Tajima F (1993). Simple methods for testing the molecular evolutionary clock hypothesis. Genetics.

[75] Baker PJ, Britton KL, Rice DW, Rob A, Stillman TJ (1992). Structural consequences of sequence patterns in the fingerprint region of the nucleotide binding fold - implications for nucleotide specificity. J Mol Biol.

[76] Yamaguchi H, Kanayama Y, Yamaki S (1994). Purification and properties of NAD-dependent Sorbitol Dehydrogenase from apple fruit. Plant Cell Physiol.

[77] Yennawar H, Moller M, Gillilan R, Yennawar N (2011). X-ray crystal structure and small-angle X-ray scattering of sheep liver sorbitol dehydrogenase. Acta Crystallogr D.

[78] Gu ZL, Nicolae D, Lu HHS, Li WH (2002). Rapid divergence in expression between duplicate genes inferred from microarray data. Trends Genet.

[79] Wagner A (2000). Decoupled evolution of coding region and mRNA expression patterns after gene duplication: implications for the neutralist-selectionist debate. P Natl Acad Sci USA.

[80] Toufighi K, Brady SM, Austin R, Ly E, Provart NJ (2005). The botany array resource: e-northerns, expression angling, and promoter analyses. Plant J.

[81] Fasoli M, Dal Santo S, Zenoni S, Tornielli GB, Farina L, Zamboni A (2012). The grapevine expression atlas reveals a deep transcriptome shift driving the entire plant into a maturation program. Plant Cell.

[82] Sweetman C, Wong DCJ, Ford CM, Drew DP (2012). Transcriptome analysis at four developmental stages of grape berry (*Vitis vinifera cv. Shiraz*) provides insights into regulated and coordinated gene expression. BMC Genomics.

[83] Conde A, Regalado A, Rodrigues D, Costa JM, Blumwald E, Chaves MM (2015). Polyols in grape berry: transport and metabolic adjustments as a physiological strategy for water-deficit stress tolerance in grapevine. J Exp Bot.

[84] Mazzitelli L, Hancock RD, Haupt S, Walker PG, Pont SDA, McNicol J (2007). Co-ordinated gene expression during phases of dormancy release in raspberry (*Rubus idaeus L.*) buds. J Exp Bot.

[85] Pilati S, Perazzolli M, Malossini A, Cestaro A, Dematte L, Fontana P (2007). Genome-wide transcriptional analysis of grapevine berry ripening reveals a set of genes similarly modulated during three seasons and the occurrence of an oxidative burst at veraison. BMC Genomics.

[86] Fortes AM, Agudelo-Romero P, Silva MS, Ali K, Sousa L, Maltese F (2011). Transcript and metabolite analysis in *Trincadeira* cultivar reveals novel information regarding the dynamics of grape ripening. BMC Plant Biol.

[87] Melino VJ, Soole KL, Ford CM (2009). A method for determination of fruit-derived ascorbic, tartaric, oxalic and malic acids, and its application to the study of ascorbic acid catabolism in grapevines. Aust J Grape Wine Res.

[88] Wong DCJ, Sweetman C, Drew DP, Ford CM (2013). VTCdb: a gene co-expression database for the crop species *Vitis vinifera* (grapevine). BMC Genomics.

[89] Wong DCJ, Sweetman C, Ford CM (2014). Annotation of gene function in citrus using gene expression information and co-expression networks. BMC Plant Biol.

[90] Obayashi T, Okamura Y, Ito S, Tadaka S, Aoki Y, Shirota M (2014). ATTED-II in 2014: evaluation of gene coexpression in agriculturally important plants. Plant Cell Physiol.

[91] Foyer CH, Noctor G (2011). Ascorbate and glutathione: the heart of the redox hub. Plant Physiol.

[92] Wheeler GL, Jones MA, Smirnoff N (1998). The biosynthetic pathway of vitamin C in higher plants. Nature.

[93] Cramer GR, Van Sluyter SC, Hopper DW, Pascovici D, Keighley T, Haynes PA (2013). Proteomic analysis indicates massive changes in metabolism prior to the inhibition of growth and photosynthesis of grapevine (*Vitis vinifera L.*) in response to water deficit. BMC Plant Biol.

[94] Krasensky J, Jonak C (2012). Drought, salt, and temperature stress-induced metabolic rearrangements and regulatory networks. J Exp Bot.

[95] Conde A, Chaves MM, Geros H (2011). Membrane transport, sensing and signaling in plant adaptation to environmental stress. Plant Cell Physiol.

[96] Larkin MA, Blackshields G, Brown NP, Chenna R, McGettigan PA, McWilliam H (2007). Clustal W and Clustal X version 2.0. Bioinformatics.

[97] Tamura K, Stecher G, Peterson D, Filipski A, Kumar S (2013). MEGA6: molecular evolutionary genetics analysis Version 6.0. Mol Biol Evol.

[98] Nei M, Kumar S (2000). Molecular Evolution and Phylogenetics.

[99] Jones DT, Taylor WR, Thornton JM (1992). The rapid generation of mutation data matrices from protein sequences. Comput Appl Biosci.

[100] Altschul SF, Gish W, Miller W, Myers EW, Lipman DJ (1990). Basic local alignment search tool. J Mol Biol.

[101] Wang YP, Tang HB, DeBarry JD, Tan X, Li JP, Wang XY (2012). MCScanX: a toolkit for detection and evolutionary analysis of gene synteny and collinearity. Nucleic Acids Res.

[102] Abagyan R, Totrov M (1994). Biased probability Monte-Carlo conformational searches and electrostatic calculations for peptides and proteins. J Mol Biol.

[103] Reimand J, Arak T, Vilo J (2011). g: profiler-a web server for functional interpretation of gene lists (2011 update). Nucleic Acids Res.

