# Supplementary figures and images for: New insights into the evolutionary history of plant sorbitol dehydrogenase
Source: BMC Plant Biol. 2015 Apr 12;15:101. doi: 10.1186/s12870-015-0478-5 (PMC4404067; doi:10.1186/s12870-015-0478-5)

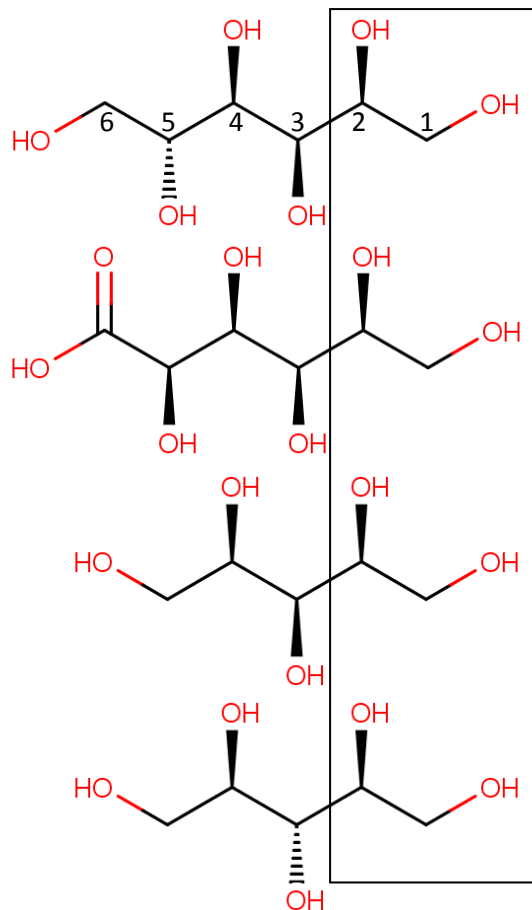

**A. Sorbitol**

**B. L-idonic acid**

**C. Xylitol**

**D. Ribitol**

Supplement: Additional file 1: — Displays the molecular structures of SDH substrates. [file 12870_2015_478_MOESM1_ESM.pdf]

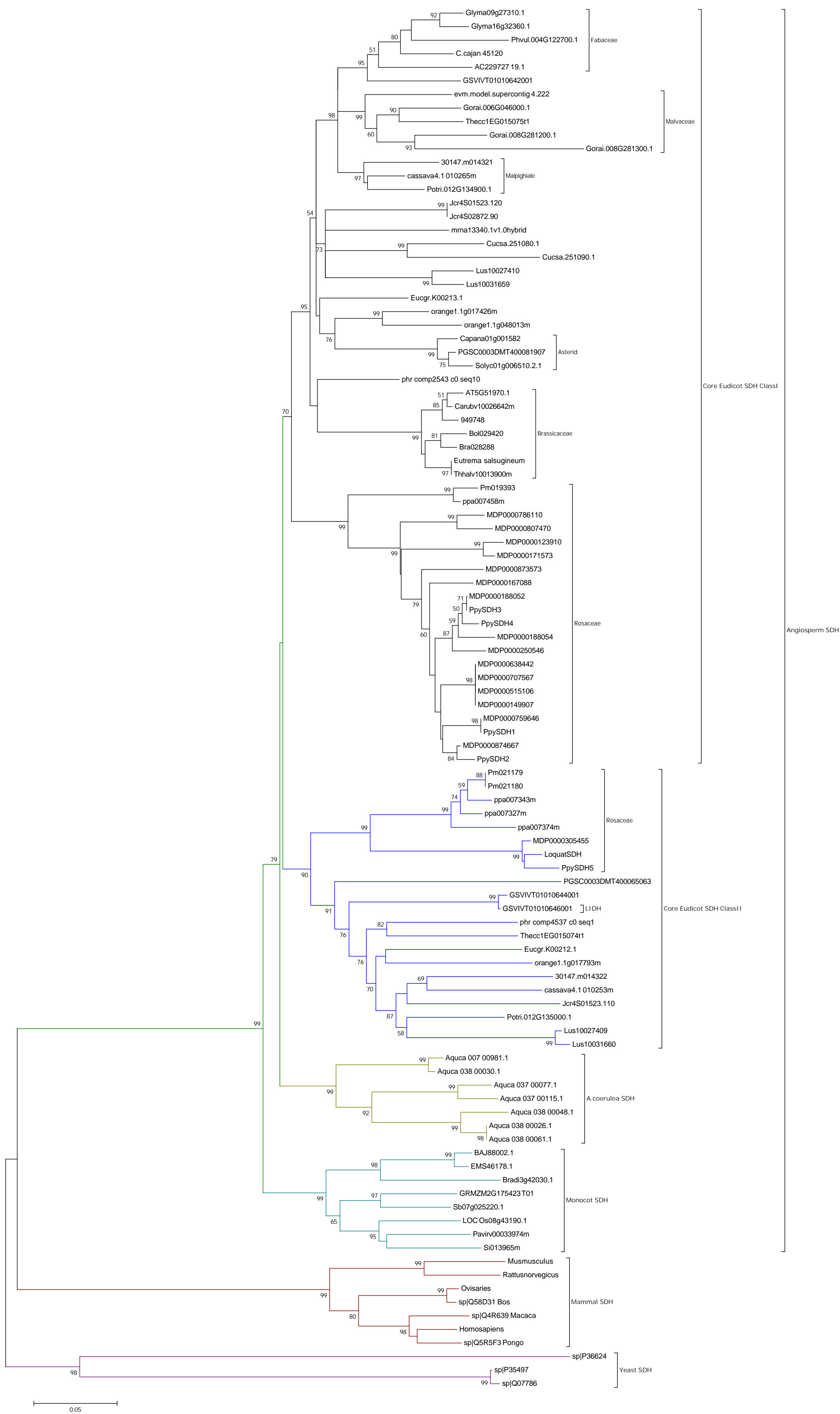

Supplement: Additional file 4: — Displays the complete Neighbour Joining tree for Figure 2 A. [file 12870_2015_478_MOESM4_ESM.pdf]

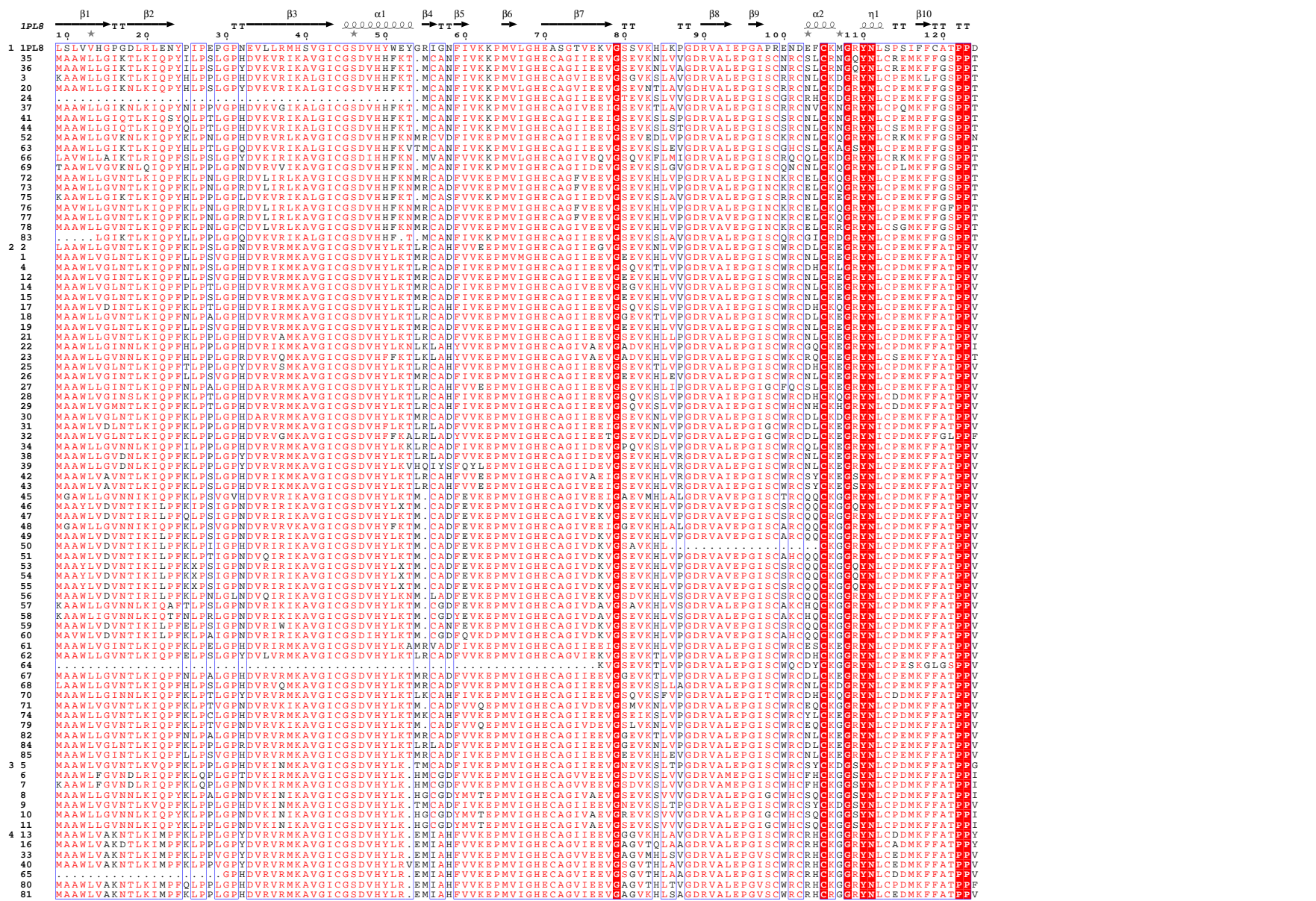



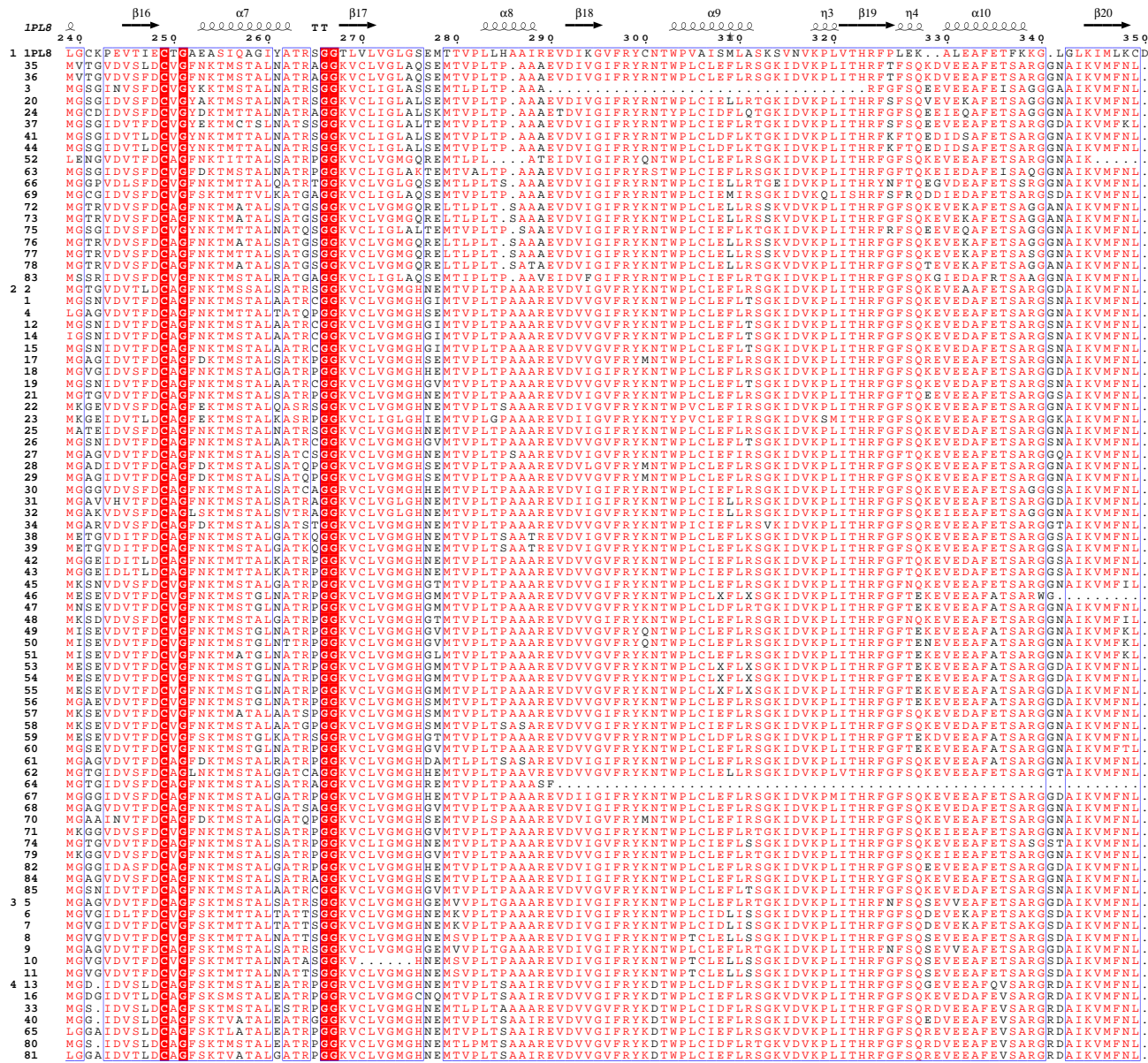

Supplement: Additional file 5: — Displays complete sequence alignment for Figure 3. [file 12870_2015_478_MOESM5_ESM.pdf]
